# Ecological Momentary Assessment of Mental Health Problems Among University Students: Data Quality Evaluation Study

**DOI:** 10.2196/55712

**Published:** 2024-12-10

**Authors:** Ana Portillo-Van Diest, Philippe Mortier, Laura Ballester, Franco Amigo, Paula Carrasco, Raquel Falcó, Margalida Gili, Glenn Kiekens, Francisco H Machancoses, Jose A Piqueras, Marisa Rebagliato, Miquel Roca, Tíscar Rodríguez-Jiménez, Jordi Alonso, Gemma Vilagut

**Affiliations:** 1 Hospital del Mar Research Institute Barcelona Spain; 2 Centro de Investigación Biomédica en Red (CIBER) de Epidemiología y Salud Pública Instituto de Salud Carlos III Madrid Spain; 3 Department of Medicine and Life Sciences Universitat Pompeu Fabra Barcelona Spain; 4 Department of Medicine Science Health Faculty Universitat Jaume I Castelló de la Plana Spain; 5 Epidemiology and Environmental Health Joint Research Unit Fundació per al Foment de la Investigació Sanitària i Biomèdica de la Comunitat Valenciana (FISABIO)-Universitat Jaume I-Universitat de València Valencia Spain; 6 Department of Education Sciences University of La Rioja Logroño Spain; 7 Instituto Universitario de Investigación en Ciencias de la Salud-Instituto de Investigación Sanitaria Illes Balears (IUNICS-IDISBA) University of Balearic Islands Palma de Mallorca Spain; 8 Department of Psychology University of Balearic Islands Palma de Mallorca Spain; 9 Center for Contextual Psychiatry KU Leuven Leuven Belgium; 10 Research Unit of Clinical Psychology KU Leuven Leuven Belgium; 11 Department of Medical and Clinical Psychology Tilburg University Tilburg Netherlands; 12 Department of Health Psychology Miguel Hernandez University of Elche Elche Spain; 13 Department of Psychology and Sociology University of Zaragoza Teruel Spain

**Keywords:** experience sampling method, ecological momentary assessment, mental health, university students, participation, compliance, reliability, sensitivity analysis, mobile phone

## Abstract

**Background:**

The use of ecological momentary assessment (EMA) designs has been on the rise in mental health epidemiology. However, there is a lack of knowledge of the determinants of participation in and compliance with EMA studies, reliability of measures, and underreporting of methodological details and data quality indicators.

**Objective:**

This study aims to evaluate the quality of EMA data in a large sample of university students by estimating participation rate and mean compliance, identifying predictors of individual-level participation and compliance, evaluating between- and within-person reliability of measures of negative and positive affect, and identifying potential careless responding.

**Methods:**

A total of 1259 university students were invited to participate in a 15-day EMA study on mental health problems. Logistic and Poisson regressions were used to investigate the associations between sociodemographic factors, lifetime adverse experiences, stressful events in the previous 12 months, and mental disorder screens and EMA participation and compliance. Multilevel reliability and intraclass correlation coefficients were obtained for positive and negative affect measures. Careless responders were identified based on low compliance or individual reliability coefficients.

**Results:**

Of those invited, 62.1% (782/1259) participated in the EMA study, with a mean compliance of 76.9% (SD 27.7%). Participation was higher among female individuals (odds ratio [OR] 1.41, 95% CI 1.06-1.87) and lower among those aged ≥30 years (OR 0.20, 95% CI 0.08-0.43 vs those aged 18-21 years) and those who had experienced the death of a friend or family member in the previous 12 months (OR 0.73, 95% CI 0.57-0.94) or had a suicide attempt in the previous 12 months (OR 0.26, 95% CI 0.10-0.64). Compliance was particularly low among those exposed to sexual abuse before the age of 18 years (exponential of β=0.87) or to sexual assault or rape in the previous year (exponential of β=0.80) and among those with 12-month positive alcohol use disorder screens (exponential of β=0.89). Between-person reliability of negative and positive affect was strong (R_kRn_>0.97), whereas within-person reliability was fair to moderate (R_cn_>0.43). Of all answered assessments, 0.86% (291/33,626) were flagged as careless responses because the response time per item was <1 second or the participants gave the same response to all items. Of the participants, 17.5% (137/782) could be considered careless responders due to low compliance (<25/56, 45%) or very low to null individual reliability (raw Cronbach α<0.11) for either negative or positive affect.

**Conclusions:**

Data quality assessments should be carried out in EMA studies in a standardized manner to provide robust conclusions to advance the field. Future EMA research should implement strategies to mitigate nonresponse bias as well as conduct sensitivity analyses to assess possible exclusion of careless responders.

## Introduction

### Background

Cross-sectional and traditional longitudinal study designs, which conduct assessments at one point or across several time points (usually 2-5 surveys months to years apart), have been widely used in the field of mental health epidemiology [1,2]. These methods serve well to provide detailed information on a population’s mental health at a given time and present important insights into epidemiological patterns (eg, prevalence, incidence, and persistence rates). They are also able to identify who within the population under study is more at risk of having mental health problems by assessing risk and protective factors that are static in the middle to long term, such as sociodemographic characteristics, personality traits, or childhood experiences. However, it is known that mental health outcomes are complex and key symptoms and risk factors such as stress, affect, sleep, and suicidal ideation may fluctuate in a matter of hours or even minutes [3-7]. Thus, it is important to study not just who is at risk but also *when* and under what daily life conditions individuals may be more vulnerable in the short term. This has prompted a diversification in how mental health is studied by introducing high-resolution designs [8]. Ecological momentary assessment (EMA; also referred to as “experience sampling method” [ESM]) is a type of intensive longitudinal study that collects survey data several times a day over multiple days [9], providing information on variables as they fluctuate and unfold in the context of daily life [10]. These data allow for the development of explanatory and predictive models at both an individual and group level [8] and enable individualized and contextualized interventions at critical moments when the risk of mental health problems may be high [3,11,12]. To date, these studies have typically relied on small sample sizes using nonrepresentative sampling designs [7,13], leaving important questions about EMA data representativeness and reliability unaddressed.

Although methodological guidelines have emerged in recent years [7,14,15], there is a large heterogeneity of study designs as well as an underreporting of methodological details and data quality parameters [16,17]. Hence, to avoid methodological anarchy, it is important that studies provide a thorough description of the methodology and basic information about EMA data representativeness in terms of degree and determinants of participation and compliance [16,18]. Providing this information is fundamental for ensuring the validity, reliability, and generalizability of study findings and improving the overall quality of the research. Furthermore, there needs to be an evaluation of the reliability of the measures as well as the participants to assess whether the collected data are robust enough to provide solid results. Assessment of the reliability of the measures in EMA data provides information on how reliable scales are to capture differences between and within individuals [14,19]. Unreliable answers are those that have been provided without paying sufficient attention to the questions, what has been commonly described as “careless responding” [20-22], and can be evaluated in 2 ways. A priori items can be used that either explicitly ask participants whether they have paid attention while answering the assessments or test the participants’ level of attention [23,24]. Alternatively, post hoc analyses have been proposed to detect possible careless responding by looking at parameters such as duration of response or variance in responses [20,21,24].

### Objectives

It has been well established that the university years are a sensitive period for the onset and persistence of mental health problems [25-27], and there has been a growing interest in the use of EMA among university students [28-30]. To our knowledge, no large-scale epidemiological study has provided an in-depth analysis of the representativeness and reliability of EMA data among university students. In this paper, we present data collected as part of the Promoting Mental Health Among University Students (PROMES-U) project, in which undergraduate students from 5 public universities in Spain were invited to participate in a web-based survey and subsequent 15-day EMA study [31]. This study addresses this gap in the literature by assessing EMA data quality in complementary ways. First, we estimate the participation rate and mean compliance and identify predictors of individual-level participation and compliance. Second, we provide between- and within-person reliability scores for measures of negative affect (NA) and positive affect (PA). Third, we build on previous work to identify potential careless responding [20,21].

## Methods

### Study Design and Population

The PROMES-U project is a prospective observational multicenter cohort study implementing 2 web-based self-reported surveys (one at baseline and one at the 12-month follow-up) and a 15-day EMA study and is part of the World Health Organization World Mental Health International College Student Initiative [32]. The project’s target population consists of undergraduate students from 5 public universities in Spain: University of the Balearic Islands, Jaume I University, Miguel Hernández University, Pompeu Fabra University, and University of Zaragoza. All Spanish-speaking students aged ≥18 years enrolled in any of the participating universities were eligible to participate. The 15-day EMA study immediately followed the baseline web-based survey (April 2022-June 2022) with asynchronous and continuous enrollment and is the focus of this report.

For further details regarding the PROMES-U project, its registration can be consulted on the internet [33].

### Recruitment

Recruitment for the baseline web survey was conducted through mass emailing using university administrative lists (census sampling), with 1 to 3 reminder emails (in 3 of the 5 participating universities and in some colleges of a fourth one) as well as on-campus project information sessions, classroom announcements, links on the universities’ websites, poster campaigns, and social media posts on the universities’ accounts. A total of 2427 eligible students completed the baseline survey (operationalized as having provided basic sociodemographic variables, >50% of the required questions, and the mental health section of the survey; Multimedia Appendix 1).

Recruitment for the 15-day EMA study was done using quota sampling based on 30-day mental disorder screens in the baseline web survey. Students were hierarchically assigned to 1 of 4 mutually exclusive subsamples. The first subsample was composed of students who had reported passive or active suicidal ideation in the previous 30 days on the Columbia-Suicide Severity Rating Scale [34]. The second subsample consisted of students without 30-day suicidal ideation but with a positive screen for harmful alcohol use. This was assessed using core items of the Alcohol Use Disorders Identification Test scale (ie, frequency of consumption, quantity, and frequency of binge drinking episodes), which ranged from 0 (*never*) to 4 (*4 or more times a week*). Those who scored ≥4 in the sum of items screened positive for harmful alcohol use [35]. Students in the third subsample did not report 30-day suicidal ideation or harmful alcohol use but presented core symptoms of depression or anxiety in the previous 30 days through questions from the Composite International Diagnostic Interview screening scales [36]. Core symptoms of depression were “feeling sad or depressed,” “feeling discouraged about how things were going in their life,” and “taking little or no interest or pleasure in things,” which were operationalized as indicating “most of the time” or more. Core anxiety symptoms were “feeling worried or anxious,” “worrying about a number of different aspects in your life, such as your work, family, health, or finances,” “feeling more worried than other people in your same situation,” and “worrying excessively or too much,” which were operationalized as indicating “most of the time” or more. Participants in the third subsample needed to present at least one of the aforementioned key symptoms of depression or anxiety. Finally, the fourth subsample (control) was composed of students who did not meet any of the aforementioned criteria.

To be invited to the EMA study, students had to complete the baseline web-based survey and provide answers for the 30-day mental disorder screens required to enable subsample assignation during quota sampling. After providing informed consent, participants were given instructions on how to participate in the study through an explainer video and link to the study’s website, as well as information on how to download and register in the ExpiWell smartphone-based app available for Android and iOS.

A total of 1259 eligible students were invited, of whom 782 (62.1%) participated: 167 (21.4%) from the suicidal ideation subsample, 234 (29.9%) from the alcohol use subsample, 206 (26.3%) from the anxiety or depression subsample, and 175 (22.4%) from the control subsample. Multimedia Appendix 1 provides more information regarding the stages of EMA study recruitment.

Almost all participants (717/782, 91.7%) began the EMA study the same day they completed the baseline web-based survey, and 99.2% (776/782) began within a week of completing the baseline survey. The length of the EMA study was suggested by stakeholders in focus groups that were carried out during the design of the project [31] and by students who participated in a pilot study and falls within what is average for EMA studies in youth [18,37].

### EMA Study Protocol and Measures

The 15-day EMA study was conducted following the baseline survey and assessed relevant constructs related to stress sensitivity (ie, affect reactivity) and mental health problems among university students. Measures were selected based on an extensive literature review by our research team and were adapted to our study’s objectives using semirandom time intervals and recall periods to decrease participant burden. Participants received push notifications on their smartphones at a random time within 2-hour time intervals in the morning (8-10 AM), midday (noon-2 PM), afternoon (4-6 PM), and evening (8-10 PM). A reminder notification was sent after 30 minutes. The median response time was 15 minutes after receiving the prompt. The momentary assessment was marked as missed if the participant did not complete the assessment within the 2-hour window.

On days 1 and 15, the instrument evaluated 2-week major depressive disorder using the 9-item Patient Health Questionnaire [38], 2-week generalized anxiety disorder using the 7-item Generalized Anxiety Disorder scale [39], and 2-week mental well-being using the Short Warwick-Edinburgh Mental Wellbeing Scale [40].

On days 2 to 15, participants were asked to complete 4 momentary assessments each day. Each assessment included twelve 7-point Likert-type scales (from *not at all* to *a lot*) that consisted of an introductory phrase (“Right now, I feel...”) followed by very short descriptions of momentary states. They were used to assess stress levels (“...stress”) from the ESM item repository [41] and energy levels (“...tired, with little energy”) and perceived concentration capacity (“...having trouble concentrating”) from the 9-item Patient Health Questionnaire [38].

Affect was assessed using 9 of the described 7-point Likert-type scales based on the 2-axis circumplex model of affect [42] (ie, high or low, and positive or negative valence). All items assessing affect were taken from validated scales, namely, the PHQ-9 [38], the 7-item Generalized Anxiety Disorder scale [39], the Short Warwick-Edinburgh Mental Wellbeing Scale [43], and the ESM item repository [41]. In total, 5 items were used to measure PA (ie, feeling “happy,” “interest in doing things,” “relaxed, “optimistic,” or “pleasure in doing things”) and 4 to measure NA (ie, feeling “nervous,” “upset,” “depressed,” or “worried”). PA and NA scores for each individual momentary assessment were calculated as the person-specific mean of the corresponding items.

Finally, 3 additional multiple-response items taken from the ESM item repository assessed momentary social context (company), physical context (location), and activity [41]. Morning and evening assessments contained additional questions regarding the previous night’s sleep duration and quality, as well as alcohol use, type of stressful events, and suicidal ideation within the previous 24 hours [41,44-47]. More information regarding the EMA measures used in this study is provided in Multimedia Appendix 2.

### Explanatory Variables for Participation and Compliance

Explanatory variables for partic9ipation and compliance were collected in the baseline web survey, which was developed by the World Mental Health International College Student Initiative consortium and implemented using the Qualtrics survey platform (Qualtrics International Inc) [31,48]. In this survey, we included four groups of explanatory variables: (1) sociodemographic and college-related variables, (2) childhood and adolescence adverse experiences, (3) stressful events in the previous 12 months, and (4) mental disorder screens for the previous year. Multimedia Appendix 3 provides more information regarding the web-based survey measures.

Sociodemographic and college-related variables included sex, nationality, age, and field of study. Childhood and adolescence adverse experiences before the age of 18 years were assessed using 19 items adapted from several questionnaires. Items that assessed parental psychopathology (ie, parent with any serious mental or emotional problems, substance abuse, suicidal thoughts and behaviors or death by suicide, and criminal activities or interpersonal violence) were taken from the Adverse Childhood Experiences Study survey [49]. Items measuring neglect and sexual and emotional abuse were from the Childhood Trauma Questionnaire–Short Form [50]. Physical abuse was reported using items from the Childhood Trauma Questionnaire–Short Form and the Army Study to Assess Risk and Resilience in Servicemembers New Soldier Study survey [51]. Bully victimization (ie, direct verbal or physical bullying as well as indirect bullying and cyberbullying) was measured using items of the Youth Risk Behavior Survey [52]. Finally, dating violence was measured using items from the 2016 to 2017 Healthy Minds Study [53].

Past-year stressful events were assessed using 11 dichotomous items based on the Army Study to Assess Risk and Resilience in Servicemembers survey [54] that included life-threatening illness or injury of a friend or family member; death of a friend or family member; breakup with a romantic partner; cheating of a romantic partner; serious betrayal by someone close; serious ongoing arguments or breakup with friends or family members; and having had a life-threatening accident or illness, been physically assaulted, experienced sexual harassment, been sexually assaulted or raped, or had a serious legal problem (ie, any serious legal problem or problems with the police).

We evaluated major depressive disorder and generalized anxiety in the previous 12 months using the Composite International Diagnostic Interview screening scales [36]. We evaluated alcohol use disorder in the previous 12 months using the Alcohol Use Disorders Identification Test [55]. We assessed suicidal thoughts and behaviors in the previous 12 months using an adapted version of the Columbia-Suicide Severity Rating Scale [34,56].

### Statistical Analysis

#### EMA Study Sample Representativity

First, we assessed the representativity of the EMA sample vis-à-vis the target population by comparing the distribution of sociodemographic and college-related characteristics of the sample during various stages of EMA study recruitment on the one hand with the distribution of these characteristics in the target populations on the other hand. Target populations were defined in two ways: (1) all students in Spanish public universities, with information on key characteristics obtained from the Ministry of Education, Vocational Training, and Sports [57]; and (2) all students in the participating universities, with the information being obtained from the universities themselves.

#### Predictors of EMA Study Participation and Compliance

EMA study participation rate and mean compliance were calculated for the total sample and stratified by the explanatory variables under study (refer to the Explanatory Variables for Participation and Compliance section). Participation rate was defined as the number of respondents who provided a usable response divided by the total number of initial personal invitations requesting participation (ie, participants who completed at least one momentary assessment divided by those who were invited to participate in the EMA study [58]). Compliance for each participant was defined as the percentage of momentary assessments completed by the participant out of the maximum number of momentary assessments allowed by the design [16]—in our case, a maximum of 56 assessments. Mean compliance was calculated from the individual compliance rates of all EMA study participants. The associations between the explanatory variables and participation were assessed using logistic regression, whereas Poisson regression was used to assess associations between the explanatory variables and compliance. Separate models were constructed for each explanatory variable, each time adjusting for all sociodemographic and college-related variables. Results are presented as odds ratios (ORs) for logistic regression models and as the exponential of the β coefficient for Poisson regression models, with corresponding 95% CIs.

#### Variance and Reliability of Measures

The overall variance of PA and NA scores was broken down into between-person and within-person variances using a multilevel model. Between-person variance measures the variance of the measure due to differences observed between participants, whereas within-person variance accounts for the variance due to differences in scores of that measure within a participant. Intraclass correlation coefficients (ICCs) for PA and NA were then calculated by dividing the between-person variance by the sum of the between- and within-person variance [14]. The ICC was calculated for the full sample of EMA study participants (n=782) as well as separately for the control subsample (n=175) and the subsample of participants with mental health problems in the previous 30 days (n=607). The ICC is a useful measure to assess whether the construct to be assessed changes within a person in such a way that its frequent assessment is justified. An ICC of <0.5 means that variance is mostly due to within-person differences (ie, momentary fluctuations). The *ggplot* and *lattice* packages in R (R Foundation for Statistical Computing) were used to visualize within-person variability for PA and NA in 7 random individuals within each subsample (28 in total).

We evaluated between- and within-person reliability of PA and NA measures using a multilevel mixed-effects model in which items and time were treated as random effects, with items nested within assessments that were then nested within persons [59]. Between-person reliability, obtained using the R_kRn_ estimate developed by Shrout and Lane [60], reflects the internal consistency reliability of between-person differences in the set of items that form the PA and NA scale scores. On the other hand, within-person reliability was obtained using the R_cn_ estimate [60], which assesses the internal consistency reliability of the set of PA and NA items across time within individuals [61]. These indicators range from 0 to 1 and can be interpreted using classic test theory conventions (0.00-0.10=practically no reliability; 0.11-0.40=slight reliability; 0.41-0.60=fair reliability; 0.61-0.80=moderate reliability; 0.81-1.00=substantial reliability) [62]. Raw Cronbach α reliabilities were calculated for each participant for the PA and NA items across time. Reliability estimates were obtained using the *mlr* function from the *psych* R package (version 2.2.9) [59].

#### Careless Responding

We evaluated careless responding by expanding on previous work by Jaso et al [21], which provides a data-driven approach and makes a distinction between careless responses and careless responders. They identify “careless responses” by evaluating how the relationship between psychometric antonyms (ie, items with opposite meaning) is affected at different thresholds of three parameters: (1) an overly fast time to complete the assessment, (2) an overly narrow within-assessment response variance, and (3) a high percentage of items with the same response. The premise of the method is that, when the relationship between the psychometric antonyms becomes neutral or positive, the response can be considered as careless. Although we followed the method by Jaso et al [21], we did not use the R package they developed as it was not applicable due to differences in measurements. For example, the variables in the study by Jaso et al [21] were measured using sliders that ranged from 0 to 100 with a default answer at 50, whereas our instrument consisted of obligatory checkbox questions ranging from 1 to 7 with no default answers.

In line with the work by Jaso et al [21], psychometric antonyms were determined by selecting the 2 items that had the strongest negative correlation (ie, feeling “nervous,” “anxious,” “on edge” and feeling “relaxed”; Multimedia Appendix 4). The parameter used to evaluate whether an assessment had been completed too fast was the time per item (ie, the average number of seconds per item that it took a participant to complete a specific momentary assessment). This was calculated by dividing the total time spent completing the assessment by the number of items of that assessment. The SD of item responses and percentage of items with the same response was calculated for each momentary assessment for the conjoint set of 12 items common to all momentary assessments, all measured using a 7-point Likert-type scale assessing momentary stress, affect, depression, anxiety, and well-being (refer to the EMA Study Protocol and Measures section). Different thresholds were evaluated for each of the 3 careless responding parameters using graphs that displayed the relationship between the scores for feeling “nervous,” “anxious,” “on edge” and feeling “relaxed” at each of the thresholds.

Participants were flagged as possible “careless responders” under 2 circumstances. The first was if they had less than the minimum number of noncareless assessments needed to have a stable estimate of the mean and variability of affect [21]. To identify the number of assessments required, we selected a random subset of an increasing number of assessments for each participant (2 to 40) and, for each number of randomly selected assessments, we calculated the 2-way ICC for absolute agreement comparing the mean and SD of the PA and NA scores of each random selection to the participant’s gold standard. We did not consider the participant’s gold standard to be all 56 assessments given that only 2.9% (23/782) of participants completed all 56 assessments. Instead, we considered 40 assessments to be the gold standard, which allowed us to include 78% (610/782) of the participants in this analysis. We ran the analysis 5 times, thus selecting 5 different random samples of assessments per individual. The minimum number of reliable assessments needed was determined whenever the ICC score stabilized to a value of ≥0.90 for both the mean and SD. In addition, participants were flagged as possible careless responders if they had a within-person reliability score of <0.11 (ie, practically no reliability [62]) for either PA or NA.

All analyses were conducted using R (version 4.1.1). For the reporting of this study, we followed an adapted version of the STROBE (Strengthening the Reporting of Observational Studies in Epidemiology) checklist specific for reporting EMA studies (Checklist for Reporting EMA Studies) [63]. The completed checklist can be found in Multimedia Appendix 5.

### Ethical Considerations

Ethics approval was provided by the Parc de Salut Mar Clinical Research Ethical Committee (protocol 2020/9198/I). The project complies with the principles established by national and international regulations, including the Declaration of Helsinki. Explicit informed consent was obtained at the beginning of the web-based surveys, and a separate consent form was provided at the end of the web-based survey for participation in the EMA study. Data were deidentified before all analyses through pseudoanonymization using encrypted identifiers to ensure privacy, and neither the study investigators nor the individuals conducting the analyses had access to the personal data. Participants who completed at least 80% of the momentary assessments as well as the mental health screener scales of days 1 and 15 received a monetary incentive of 30€ (US $32.03). Participants were informed in advance of the requirements to receive the incentive and were not informed explicitly when they qualified for it, nor did the app show any information on compliance other than which assessments had been completed.

## Results

### EMA Study Sample Representativity

A total of 1259 baseline survey participants were invited to take part in the EMA study, of whom 1126 (89.4%) provided informed consent, and 782 (62.1% of those invited; 69.4% of those providing informed consent) participated (Multimedia Appendix 1). Table 1 shows the distribution of sociodemographic and college-related characteristics of the target populations and of the sample across the various phases of recruitment for the EMA study up until EMA participation. Compared to the target populations, EMA participants were more likely to be women (619/782, 79.2% vs 39,214/69,805, 56.2%-753,749/1,338,304, 56.3%), younger students (577/782, 73.8% vs 724,853/1,338,304, 54.2%-43,664/69,805, 62.6%), and science (94/782, 12% vs 3633/69,805, 5.2%-84,750/1,338,304, 6.3%) and health sciences (219/782, 28% vs 258,967/1,338,304, 19.4%-14,316/69,805, 20.5%) students.

These observed differences in EMA sample composition compared to the target population are mainly due to a different likelihood of participation in the baseline survey and, to a far lesser extent, to a different likelihood of EMA participation among those invited from the baseline survey.

**Table 1:** Distribution of key sociodemographic and college-related characteristics of the ecological momentary assessment (EMA) study target student populations and of the EMA study participants across the EMA study recruitment phases.

			EMA study target population				EMA study recruitment phases						
			All public universities in Spain^a^ (n=1,338,304), n (%)		PROMES-U^b^ universities^c^ (n=69,805), n (%)		Completed baseline web survey^d^ (n=2427), n (%)		Invited to the EMA study^e^ (n=1259), n (%)		Provided IC^f^ for EMA study (n=1126), n (%)		Participated in EMA study^g^ (n=782), n (%)
**Sex**													
	Male	584,555 (43.7)		30,591 (43.8)		609 (25.1)		288 (22.9)		243 (21.6)		163 (20.8)	
	Female	753,749 (56.3)		39,214 (56.2)		1818 (74.9)		971 (77.1)		883 (78.4)		619 (79.2)	
**Nationality**													
	Spanish	1,253,880 (93.7)		65,444 (93.8)		2236 (92.1)		1156 (91.8)		1027 (91.2)		722 (92.3)	
	Other	84,424 (6.3)		4361 (6.2)		191 (7.9)		103 (8.2)		99 (8.8)		60 (7.7)	
**Age (y)**													
	18-21	724,853 (54.2)		43,664 (62.6)		1722 (71)		894 (71)		800 (71)		577 (73.8)	
	22-25	320,156 (23.9)		17,701 (25.4)		539 (22.2)		297 (23.6)		267 (23.7)		176 (22.5)	
	26-29	105,557 (7.9)		3988 (5.7)		73 (3)		36 (2.9)		33 (2.9)		21 (2.7)	
	≥30	187,738 (14)		4452 (6.4)		93 (3.8)		32 (2.5)		26 (2.3)		8 (1)	
**Field of study**													
	Arts and humanities	140,969 (10.5)		6177 (8.8)		269 (11.1)		154 (12.2)		132 (11.7)		88 (11.3)	
	Sciences	84,750 (6.3)		3633 (5.2)		217 (8.9)		141 (11.2)		121 (10.7)		94 (12)	
	Health sciences	258,967 (19.4)		14,316 (20.5)		667 (27.5)		335 (26.6)		315 (28)		219 (28)	
	Social and legal sciences	616,880 (46.1)		32,340 (46.3)		997 (41.1)		470 (37.3)		420 (37.3)		280 (35.8)	
	Engineering and architecture	236,738 (17.7)		13,339 (19.1)		277 (11.4)		159 (12.6)		138 (12.3)		101 (12.9)	

^a^Information retrieved from the Spanish Ministry of Education and Professional Formation [64].

^b^PROMES-U: Promoting Mental Health Among University Students.

^c^Information obtained from each of the 5 participating universities, accessible on demand.

^d^Defined as making it to the end of the baseline web survey, as well as having provided basic sociodemographic and college-related variables (ie, sex, nationality, age, and field of study), having completed the mental health section, and having answered >50% of the required questions.

^e^Defined as having been assigned to a group following the hierarchical quota sampling scheme (refer to the Sampling Procedures subsection in the Methods section).

^f^IC: informed consent.

^g^Defined as having completed at least one momentary assessment in the EMA study.

### Predictors of EMA Study Participation and Compliance

**Figure 1:**
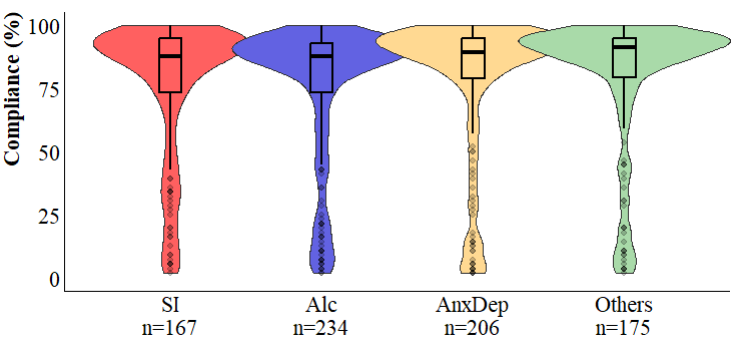
Violin plots showing the distribution of compliance (ie, the percentage of momentary assessments completed out of the maximum of 56 allowed by the design) stratified by each subsample of students: suicidal ideation, harmful alcohol use, anxiety or depression, and controls. Alc: harmful alcohol use; AnxDep: anxiety and depression; SI: suicidal ideation.

The EMA study’s participation rate was 62.1% (782/1259). Mean compliance was 76.9% (SD 27.7%; range 1.9%-100%), with a median of 89.3% and a mode of 96.4% (Figure 1). A total of 82.5% (645/782) of the participants completed at least one assessment on the final day.

Multimedia Appendix 6 shows the associations between participation and compliance and sociodemographic and college-related characteristics, childhood and adolescence adverse experiences before the age of 18 years, stressful experiences, mental disorder screens, and suicidal thoughts and behaviors in the previous 12 months.

Female individuals had significantly higher odds of participation (OR 1.41, 95% CI 1.06-1.87), whereas those who were aged ≥30 years (OR 0.20, 95% CI 0.08-1.06), had had a death of a friend or family member in the previous year (OR 0.73, 95% CI 0.57-0.94), or had had a suicide attempt in the previous year (OR 0.26, 95% CI 0.10-0.64) had significantly lower odds of participation.

Compliance was significantly lower among non-Spanish participants (exponential of β=0.94, 95% CI 0.90-0.98), older age groups (exponential of β=0.88, 95% CI 0.82-0.94 for those aged 26-29 years; exponential of β=0.86, 95% CI 0.76-0.96 for those aged ≥30 years), and students of social and juridical sciences (exponential of β=0.92, 95% CI 0.89-0.95) than among arts and humanities students. Compliance was significantly lower among those who had experienced any of the childhood and adolescence adverse experiences under study except for parental psychopathology (exponential of β range 0.87-0.98), especially among those who experienced neglect (exponential of β=0.92, 95% CI 0.89-0.94) or sexual abuse (exponential of β=0.87, 95% CI 0.82-0.92). In total, 45% (5/11) of the stressful experiences in the previous 12 months under study were negatively associated with compliance (exponential of β range 0.80-0.97), especially sexual assault or rape (exponential of β=0.80, 95% CI 0.74-0.86). Compliance was higher among those with any stressful event in the previous 12 months (exponential of β=1.03, 95% CI 1.01-1.06) and those with exactly one stressful experience in the previous 12 months (exponential of β=1.09, 95% CI 1.05-1.13, compared to none). Regarding adverse mental health, compliance was lowest among those with a positive screen for alcohol use disorder in the previous 12 months (exponential of β=0.89, 95% CI 0.85-0.93) but also significantly lower among those with positive screens for major depression in the previous 12 months (exponential of β=0.95, 95% CI 0.93-0.97), generalized anxiety (exponential of β=0.95, 95% CI 0.92-0.97), and suicidal ideation (exponential of β=0.97, 95% CI 0.95-0.99). Compliance gradually decreased throughout the 15-day EMA study in a linear fashion both in the total sample and in each separate subsample (Multimedia Appendix 7).

### Variance and Reliability of Measures

**Figure 2:**
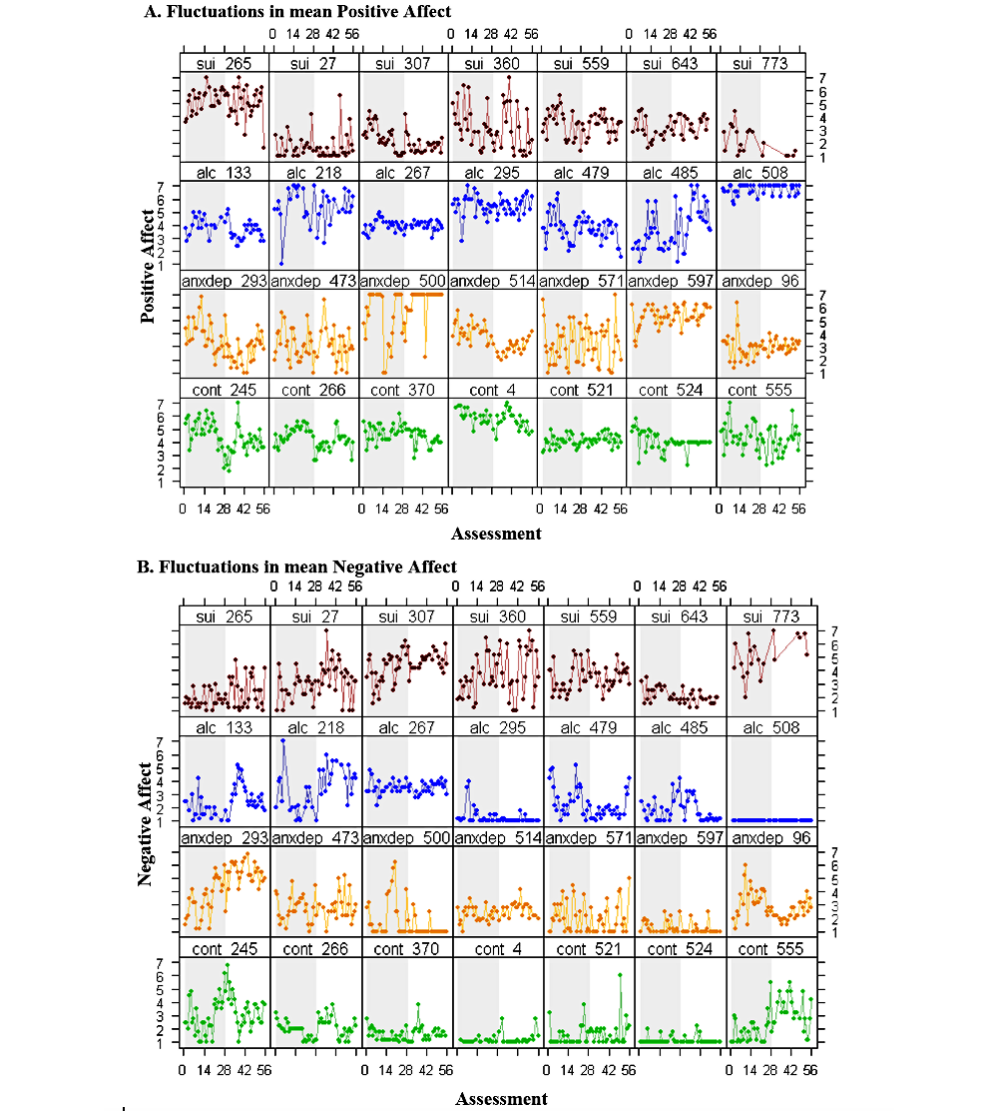
(A) Positive and (B) negative affect scores across all momentary assessments for 7 random participants from each subsample (n=28) selected among those who completed at least one assessment on the last day of the ecological momentary assessment study (n=645). Red represents the suicidal ideation subsample, blue represents the harmful alcohol use subsample, yellow represents the anxiety and depression subsample, green represents the control subsample.

Figure 2 presents PA and NA scores in all assessments for a random selection of 7 individuals within each subsample. Important differences in PA and NA scores across individuals were observed. For example, when looking at NA, some individuals presented very little intraindividual variability (eg, alc_508, anxdep_597, and cont_524), and others had important fluctuations from one assessment to the next (eg, sui_360 and anxdep_293; Figures 2A and 2B).

The total ICCs for mean PA and NA were 0.54 and 0.52, respectively. The ICC for both PA and NA was 0.50 in the subsample of participants with mental health problems, meaning that, on average, between- and within-person variance were very similar (Table 2), in line with the differences in variability observed in Figure 2. In the control subsample, the ICCs for PA and NA were slightly higher and lower (ICC=0.54 and 0.46, respectively). Overall affect variance (both between and within persons) was greater among those in the adverse mental health subsamples (Table 2).

As seen in Table 3, between-person reliability was high for both PA and NA (ie, random-effects reliability; R_kRn_=0.99). Within-person reliability was lower but still acceptable (R_cn_=0.48 and 0.59 for PA and NA, respectively). Within-person reliability was slightly higher on average among those with mental health problems. Within-person reliabilities were consistently higher for NA than for PA. Within-person reliability scores were highest when including all measured items both for PA and NA.

Multimedia Appendix 8 shows individual Cronbach α reliability coefficients by participant, which range from negative values to 0.97 for PA and 0.99 for NA. As expected, individuals that only answered 1 assessment (8/781, 1%) did not provide enough information to have reliability outcomes. Among the rest, there were 3.7% (19/782) of individuals with negative values for individual NA or PA reliability. Of all participants, 86.8% (679/782) and 66.8% (522/782) had moderate to substantial individual reliability (ie, raw Cronbach α>0.60) for PA and NA, respectively.

Participants that appeared to have very low NA variability in Figure 2B (eg, anxdep_597 and cont_524) had the lowest within-person reliability for NA (Multimedia Appendix 8). Furthermore, participant alc_508, who presented practically no variation in their mean level of reported NA, had no value for raw Cronbach α (Multimedia Appendix 8). Generally, participants with very low individual reliability scores presented either very low compliance rates or low variability in their answers.

**Table 2:** Intraclass correlation coefficients (ICCs) of positive and negative affect to show how much of their variability was due to differences between participants as opposed to fluctuations within each participant, calculated among ecological momentary assessment (EMA) participants and stratified by subsamples (N=782)^a^.

	EMA participants				Control subsample of EMA participants (n=175)				Mental health problem subsamples of EMA participants (n=607)		
	Between-person variance	Within-person variance	ICC	Between-person variance		Within-person variance	ICC	Between-person variance		Within-person variance	ICC
Positive affect	0.94	0.81	0.54	0.75		0.59	0.56	0.88		0.88	0.50
Negative affect	0.88	0.80	0.52	0.42		0.50	0.46	0.88		0.89	0.50

^a^Interpretation: an ICC of <0.5 means that the variance is mostly due to within-person differences (ie, momentary fluctuations).

**Table 3:** Multilevel between- and within-person reliability coefficients of positive affect (PA) and negative affect (NA) among ecological momentary assessment (EMA) participants stratified by subsamples (N=782).

	EMA participants		Control subsample of EMA participants (n=175)			Mental health problem subsamples of EMA participants (n=607)	
	PA items	NA items	PA items	NA items	PA items		NA items
Between-person reliability (R_kRn_^a^)	0.99	0.99	0.97	0.98	0.98		0.99
Within-person reliability (R_cn_^b^)	0.48	0.59	0.43	0.52	0.49		0.60

^a^R_kRn_: generalizability of between-person differences averaged over time; time nested within people.

^b^R_cn_: generalizability of within-person variations averaged over items; time nested within people [3].

### Careless Responses

Different thresholds were evaluated for each of the 3 careless responding parameters (ie, time per item, SD of item responses in each assessment, and percentage of items of each assessment that fell at the mode of that assessment).

**Figure 3:**
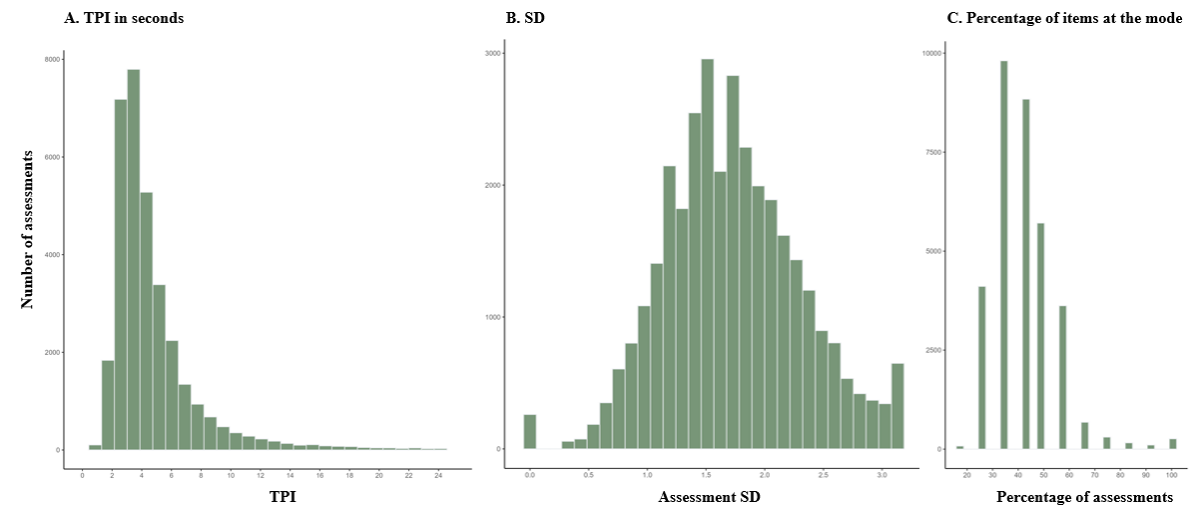
Distribution of time per item (TPI) of each assessment, SD of item responses in each assessment, and percentage of items in each assessment that fell at the mode among completed assessments (n=33,626) by ecological momentary assessment study participants (n=782).

**Figure 4:**
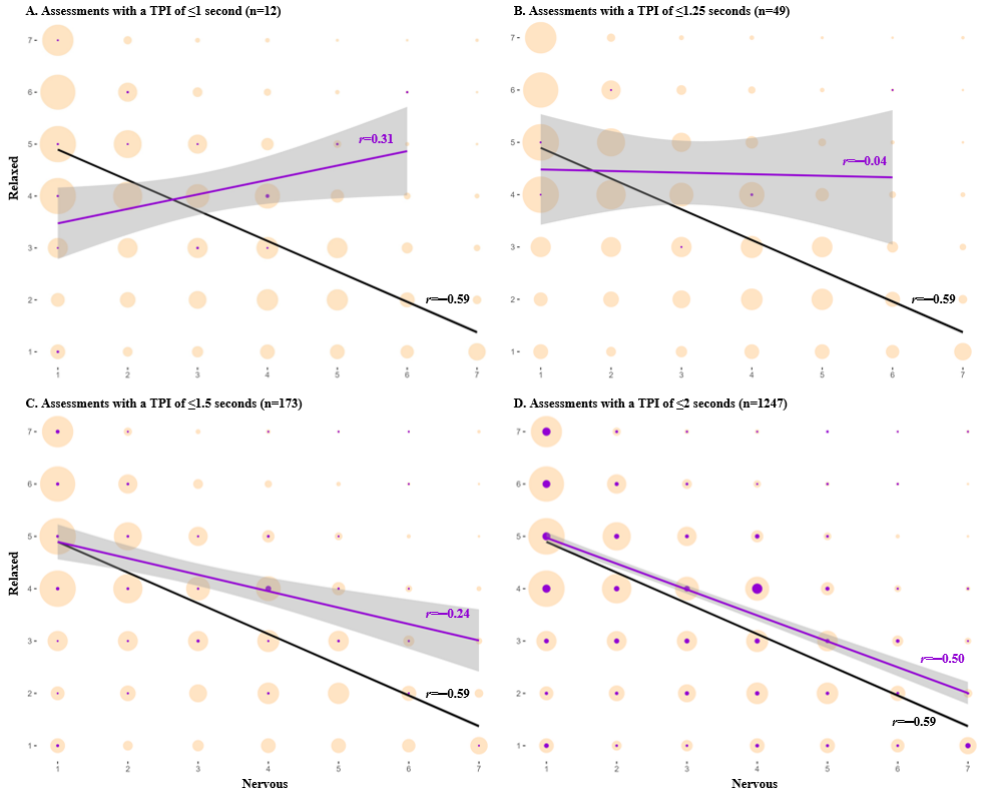
Comparing the relationship between psychometric antonym items (feeling nervous, anxious, on edge and relaxed) at different time per item (TPI) thresholds, measured in seconds, for all completed assessments (n=33,626). Purple bubbles and trendlines indicate assessments below the indicated threshold; salmon-colored bubbles and black lines indicate all answered assessments. Note: bubble sizes are relative to the number of assessments at each combination of responses to the 2 selected items.

The distribution of assessments according to time per item (Figure 3A) was sharply skewed to the right, with a median at 3.9 seconds per item. Figure 4 shows that, in assessments with a time per item of ≤1 second (*r*=−0.04), the relationship between feeling “anxious” and feeling “relaxed” flattens, thus conveying that responses below this threshold are likely careless. A total of 0.13% (44/33,626) of all completed assessments were below this threshold.

**Figure 5:**
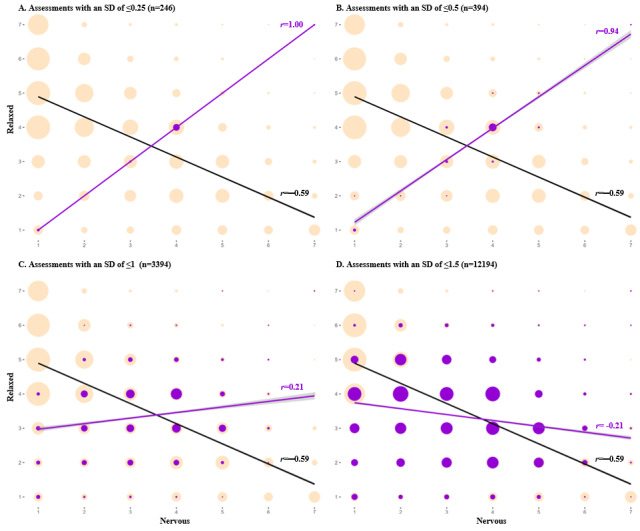
Comparing the relationship between psychometric antonym items (feeling nervous, anxious, on edge and relaxed) at different thresholds for the SD of item responses for all completed assessments (n=33,626). Purple bubbles and trendlines indicate assessments below the indicated threshold; yellow bubbles and black lines indicate all answered assessments. Note: bubble sizes are relative to the number of assessments at each point of correlation.

Figure 3B shows that the distribution of the SDs of the item responses in each assessment was normal, with a group of outliers that had an SD of 0 (indicating no variability in item responses). The relationship between psychometric antonyms shifted in those assessments at an SD of ≤1 and became bluntly positively correlated at an SD of ≤0.5 and ≤0.25 (*r*=0.94 and 1.00, respectively), which comprised 10.1%, 1.2%, and 0.7% of all answered assessments, respectively (Figure 5).

**Figure 6:**
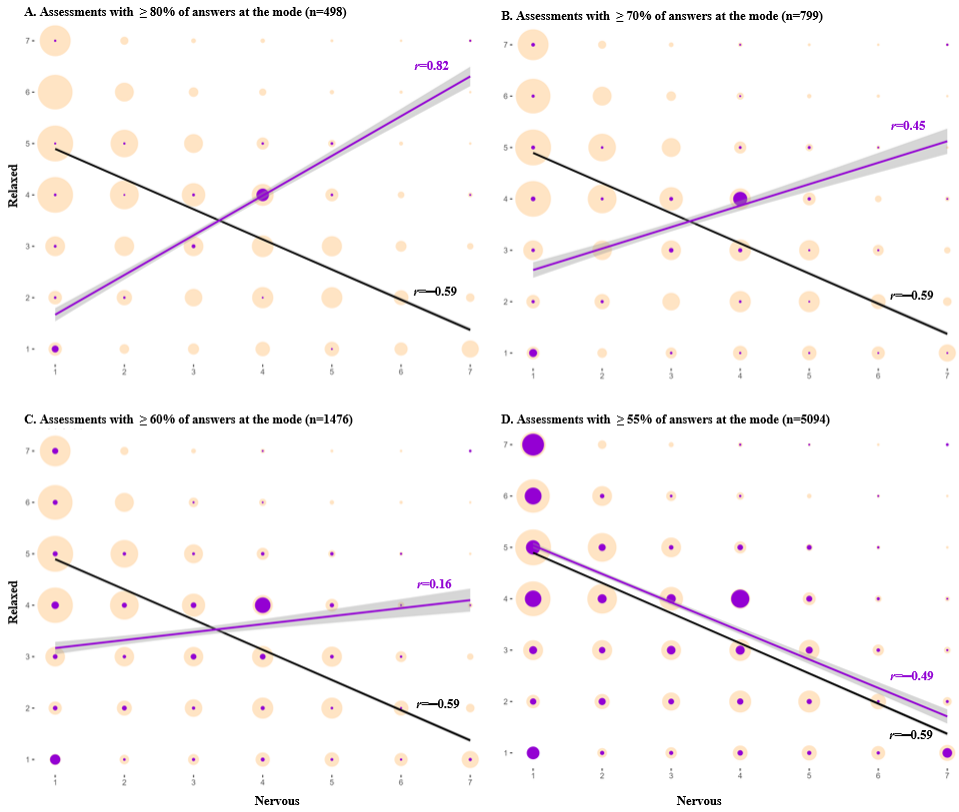
Comparing the relationship between selected psychometric antonym items (feeling nervous, anxious, on edge and relaxed) at different thresholds of percentage of items per assessment at the mode. Purple bubbles and trendlines indicate assessments below the indicated threshold; yellow bubbles and black lines indicate all answered assessments. Note: bubble sizes are relative to the number of assessments at each point of correlation.

Figure 3C shows the distribution of the percentage of individual responses within a given assessment that fall at the mode of that assessment. This distribution also had a right skew. The relationship between “anxious” and “relaxed” items flattened in assessments with ≥60% of items with answers at the mode (*r*=0.16) and became sharply positive when ≥80% of the answers fell at the mode (*r*=0.82), which comprised 4.4% and 1.5% of all answered assessments, respectively (Figure 6).

Although the relationship between the psychometric antonyms flattened when the SD was of ≤1 and when ≥60% of items with answers fell at the mode, most of the answers that were below and above these thresholds were not outliers in the distribution (Figures 3B and 3C) and were theoretically plausible responses. Thus, we decided that only assessments with no variance in responses whatsoever (ie, SD of 0 or 100% of items at the mode) should be considered careless responses. Of all the answered assessments, 0.77% (261/33,626) had no variation in responses.

Considering the aforementioned thresholds, a total of 0.86% (291/33,626) of all completed assessments were flagged as having been responded carelessly. There was little overlap between careless responses identified through no variation and those identified through a time per item of ≤1 second (Figure S3A in Multimedia Appendix 9).

When looking at how careless responses clustered within individuals, 8.8% (69/782) of participants had at least one assessment flagged as careless, 0.8% (6/782) had more than a third of their answers flagged as careless, and 0.26% (2/782) had at least half of their answers flagged as careless (Multimedia Appendix 8). Furthermore, half of all careless responses clustered around 8 participants.

To evaluate whether careless responses could be related to monetary incentives, we looked at the relationship between the number of careless responses and compliance by performing a logistic regression dichotomizing compliance at the incentive threshold (ie, 80%). We found that there were no differences in the percentage of careless responses among those who did and did not receive an incentive (OR 0.99, 95% CI 0.96-1.02; Multimedia Appendix 10).

### Careless Responders

**Figure 7:**
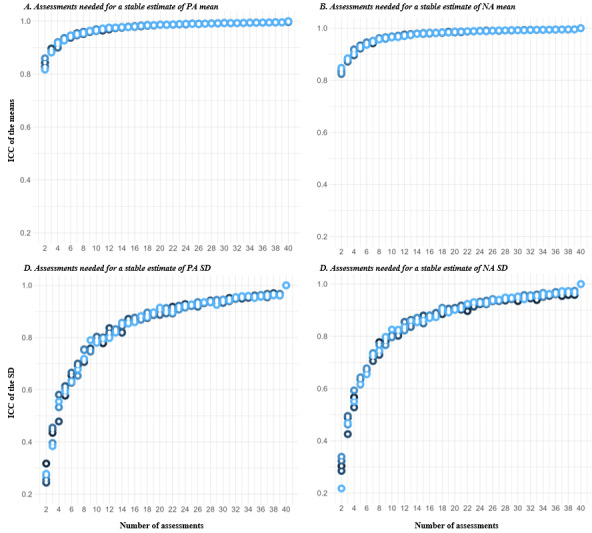
Intraclass correlation coefficient (ICC) for positive affect (PA) and negative affect (NA) score mean and SD between an increasing number of random assessments (x-axis) and its value when selecting 40 assessments (considered the gold standard) using data from all participants who completed ≥40 assessments (n=610).

Figure 7 shows the number of assessments needed to have a stable estimate of the mean and variability of PA and NA scores. The mean PA and NA scores reached an ICC of 0.9 at approximately 4 assessments, clearly stabilizing at approximately 12 assessments, whereas approximately 25 were needed to be able to capture variability well. There was a total of 3.7% (29/782) of the participants who had <25 valid assessments (ie, completed and not flagged as carelessly responded) according to the considered parameters. In total, 6.4% (50/782) of the participants had very low or null individual reliability (raw Cronbach α<0.11) for either PA or NA.

In total, 17.5% (137/782) of the participants were identified as potential careless responders (Figure S3B in Multimedia Appendix 9).

## Discussion

### Contextualization of Principal Findings

We investigated EMA data quality in a large sample of university students that included participants who screened positive for mental health problems (607/782, 77.6%) as well as students who did not (175/782, 22.4%) by using state-of-the-art data-driven analyses to assess participation, compliance, and reliability indicators. A total of 1259 students were invited to participate in the EMA study, with a participation of 782 (62.1%) and mean compliance of 76.9% (SD 27.7%). We found that participants in our EMA study were representative of the invited population, whereas compliance was slightly different across groups of explanatory variables. The data were highly reliable to account for between-person differences in NA and PA and fair to moderate to study differences within each participant. The findings suggest that 0.86% (291/33,624) of all completed assessments were responded carelessly and that 17.5% (137/782) of the participants were potential careless responders.

Participation and its predictors are rarely investigated in EMA studies given that such analyses require having information on those who refuse to participate. This demands epidemiological study designs that include careful tracking of potential participants across recruitment phases as well as probability sampling techniques. By linking our EMA study to a large cohort study, we were able to investigate and predict EMA participation and compliance, including a wide range of relevant candidate predictor variables for these outcomes. We found that men had significantly lower rates of participation, which is a tendency across scientific studies [65]. Interestingly, the only mental health outcome that was significantly associated with lower participation was having had a suicide attempt in the previous year. This could be due to multiple reasons outside the scope of this study that could affect the desire or capacity to participate in an intensive longitudinal study [66]. It is possible that students that have attempted suicide in the past year have some with the highest levels of psychological pain and stress. The only stressful event related to lower participation was having had a friend or family member die in the previous year. Mourning is a process that is also highly distressing psychologically [67] and is more common than having recent suicide attempts, thus having potentially a higher impact on overall EMA study participation.

Mean compliance was found to be similar to what other studies have reported [16,68], including a pattern of declining compliance as the days passed [16,24,69,70]. Although we cannot empirically confirm that compliance may have been positively impacted by monetary incentives, there seems to be a relationship between the 2; we did observe an inflection point around the 80% mark of compliance, and the literature has also found that studies with monetary incentives tend to have better compliance rates [16,68]. Concerning predictors of compliance, we found that almost all childhood adversities and a significant number of recent stressful events were associated with lower compliance. However, it is important to stress that effect sizes on compliance were very low and the differences across groups were generally small. Compliance was lowest among participants that had been sexually abused during their childhood or adolescence and those who had been sexually assaulted or raped in the previous year. Childhood and recent sexual abuse have been widely identified as being associated with grave emotional distress [71-73]. Concerning mental health outcomes, positive screening for alcohol use disorder was the strongest mental health predictor for low compliance. This finding aligns with the literature, which argues that activities or behaviors such as drinking alcohol and experiencing stress, which draw attention away from participating, could result in lower compliance [66,74]. Lower compliance rates among those who have lived through extremely stressful events and those who have poor mental health can also be related to how these experiences or states can impact cognition [66].

When testing reliability, we found that between-person reliability for both PA and NA was excellent (R_kRn_=0.99), whereas within-person reliability was lower (R_cn_=0.48 and 0.59 for NA and PA, respectively). EMA studies typically do not report reliability coefficients; however, when they do, within-person reliability is lower than between-person reliability, with similar results to ours [19,75,76]. This is to be expected because within-person variability is also generally smaller than variability between individuals [77]. Although it is important to evaluate between- and within-person reliability in each study as they will vary from sample to sample, they are not typically reported in EMA studies [75]. We did find 3 EMA studies that reported affect reliability scores, all of which presented very high between-person reliability scores for PA and NA, ranging from 0.96 to 1.00, and lower within-person reliability scores. This is to be expected because within-person variability is also generally smaller than variability between individuals [77]. In total, 2 of the aforementioned EMA studies calculated within-person reliability in the same way as we did and reported very similar coefficients, ranging from 0.50 to 0.71 [19,76].

Next, we attempted to identify careless responses, finding that <1% of assessments (291/33,624, 0.86%) had been answered carelessly, whereas 1 in every 6 participants in our sample was a potential careless responder. This step, though crucial for future analyses, is typically overlooked in EMA research. Potential careless responses were evaluated building on research in this area [21] (refer to the Statistical Analysis subsection in the Methods section).

When implementing the proposed method, we realized that focusing strictly on shifts in the relationship between psychometric antonyms would lead to discarding many plausible responses. For example, it is reasonable for a participant to provide similar ratings across items measuring affect if those ratings are near the middle of the scale. This issue was also raised in the study by Jaso et al [21]. Thus, we decided to take a more conservative approach, which led to simplifying the analysis from using 3 parameters to just using 2, namely, low duration and no variability in responses (ie, straight-line responding). Interestingly, these are the 2 most common parameters mentioned in the literature [20-22]. Although the overall number of careless responses in our study was negligible, it is still crucial to identify them because they tend to cluster around a small number of participants, which in turn allows us to spot careless responders.

Assessments flagged as being answered carelessly will be considered “missed” in future studies using these data. This, in turn, raises the important question as to how missing data can hamper data quality and interpretation of EMA results [66,70], especially as we have seen that missed assessments do not occur at random and may be influenced by multiple factors. Future analyses could address this issue using full information maximum likelihood [14], imputation techniques, or dynamic structural equation modeling [78].

We believe that the parameters we used to identify potential careless responders (ie, minimum number of completed assessments and individual reliability scores) can provide a stepping stone for future studies as these parameters are data driven and grounded in previous literature [20,21,24]. Determining the minimum number of assessments needed to have a stable estimate of the key measures’ mean and variability may be particularly relevant as it is common practice in EMA studies to consider an arbitrary threshold of minimum responses [14,66]. This method was also adapted from the work by Jaso et al [21]. However, they used rank-order correlation, whereas we used the ICC as we considered it to be more statistically sound given that it also accounts for agreement between the measures. We found that a minimum of 25 valid assessments (ie, 25/56, 45% compliance in the case of our study) were needed to have a stable estimate of the mean and variability of affect.

When looking at individual reliability of PA and NA scores, we observed that most participants had moderate to substantial reliability (ie, raw Cronbach α>0.60) for both PA and NA. However, some individuals had very low or even negative values, especially for NA. This mainly happened for two reasons: (1) they had low or negative interitem covariance that conformed a construct or (2) the responses to these items very seldom varied across the 56 assessments.

When the variability of the items is small, it becomes mathematically challenging to assess reliability as it is difficult to determine whether the responses truly covary (ie, have internal consistency) [19,75,77,79]. This phenomenon has been previously described in the literature [75]. Therefore, the internal consistency reliability of students with lower variability in their affective states may be penalized even if the responses are plausible. For this reason, we decided to set a low threshold of individual reliability for careless responders, flagging participants with a very low individual reliability (<0.11; “virtually no reliability”) [62]. These potentially careless responders will not be excluded from the sample but flagged for sensitivity analyses in future studies.

### Limitations

This study has several limitations that should be noted. First, although we conducted a pilot study to ensure that the software and recruitment flow were effective, technological issues such as bugs and connectivity problems could have affected participation and compliance, although reported incidences were minimal. Second, our EMA sample is not fully representative of the target population due to relatively low baseline survey participation and due to quota sampling. Nevertheless, we have detailed mental health information and basic sociodemographic data that can help mitigate this lack of representativeness and restore it for key variables through weighting procedures. Third, although we used sound methods to identify careless responders and responses, we cannot externally validate these findings as being truly indicative of careless response behavior. In any case, we will conduct sensitivity analyses in future studies to assess the impact of excluding these participants. Finally, it is important to consider that our conclusions are specific to the context of our sample and study design and may not be generalizable to other contexts.

### Conclusions

The increase in the use of EMA in the mental health epidemiology field demands standardized ways to assess data quality to provide robust conclusions to advance the field. Our data are highly reliable to account for between-person differences in affect and fair to moderate study differences within each participant. Careless responses in our sample were minimal, and 1 out of 6 participants was found to be a potential careless responder (Textbox 1).

**Ecological momentary assessment design and quality evaluation recommendations for researchers.**

**Recommendations for researchers**

- Ecological momentary assessment studies should collect basic information from nonresponders to restore representativity post hoc as it can be affected by gender, recent stressful events, and high mental health distress, among other things.
- Researchers should be mindful that compliance is particularly affected by stressful events that have an impact on cognition and by engagement in behaviors that draw attention away from participation, such as alcohol consumption.
- Careless responses can be identified post hoc when the time of response is <1 second per item or when all answers within an assessment are identical if these include psychological antonyms.
- Potential careless responders are those who have no individual reliability (raw Cronbach α<0.11) or do not have a minimum number of valid assessments to have a stable estimate of the mean and variability of key measures.

## Appendix

Multimedia Appendix 1 Flowchart of participation in the Promoting Mental Health Among University Students project.

Multimedia Appendix 2 Ecological momentary assessment measures.

Multimedia Appendix 3 Web-based survey measures.

Multimedia Appendix 4 Correlation coefficients for all items measuring affect using all data from all ecological momentary assessment participants (n=782).

Multimedia Appendix 5 Checklist for Reporting Ecological Momentary Assessment Studies.

Multimedia Appendix 6 Associations between ecological momentary assessment study participation and mean compliance and sociodemographic characteristics, childhood and adolescence adverse experiences, recent stressful events, and mental disorders in the previous 12 months.

Multimedia Appendix 7 Percentages of missed assessments per day for all participants in the ecological momentary assessment study (n=782) and for each subsample.

Multimedia Appendix 8 Within-person reliability scores for positive and negative affect, compliance, and percentage of assessments responded carelessly for all ecological momentary assessment participants (n=782).

Multimedia Appendix 9 Overlap between the different parameters to identify careless responses and responders.

Multimedia Appendix 10 Scatterplot presenting the relationship between the percentage of identified careless responses and compliance for all participants in the ecological momentary assessment study (n=782).

## Abbreviations

EMA: ecological momentary assessment
ESM: experience sampling method
ICC: intraclass correlation coefficient
NA: negative affect
OR: odds ratio
PA: positive affect
PHQ-9: 9-item Patient Health Questionnaire
PROMES-U: Promoting Mental Health Among University Students
STROBE: Strengthening the Reporting of Observational Studies in Epidemiology

## Data Availability

The datasets generated during and analyzed during this study are available from the corresponding author on reasonable request.
