# Supplementary figures and images for: Ecological Momentary Assessment of Mental Health Problems Among University Students: Data Quality Evaluation Study
Source: J Med Internet Res. 2024 Dec 10;26:e55712. doi: 10.2196/55712 (PMC11668991; doi:10.2196/55712)

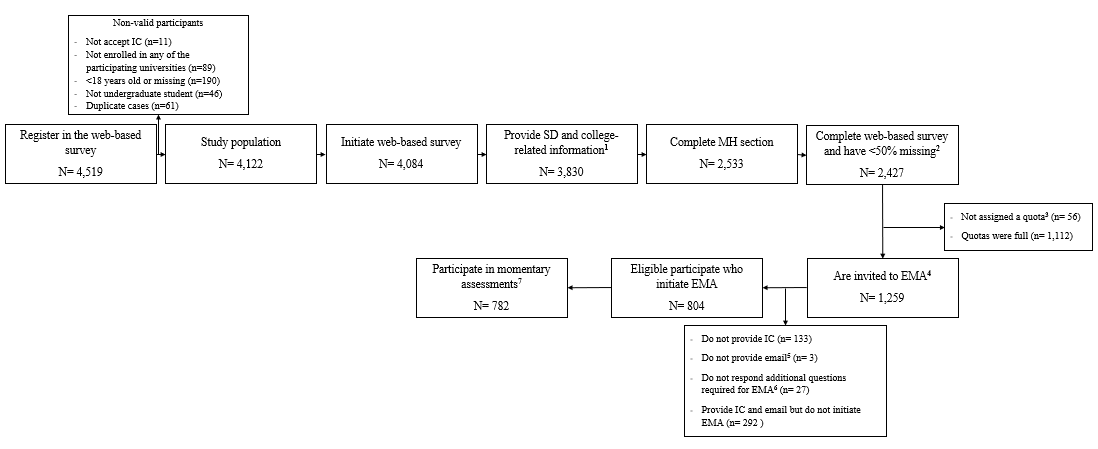

Supplement: Multimedia Appendix 1 [file jmir_v26i1e55712_app1.png]

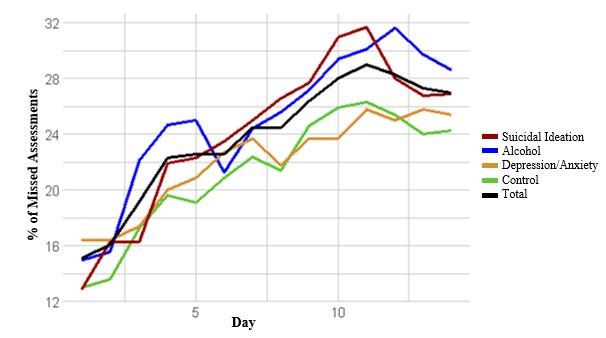

Supplement: Multimedia Appendix 7 [file jmir_v26i1e55712_app7.png]

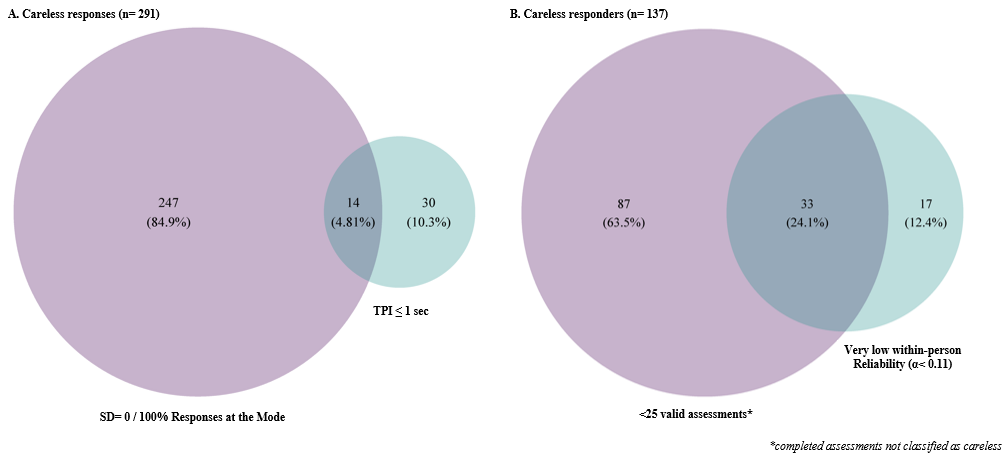

Supplement: Multimedia Appendix 9 [file jmir_v26i1e55712_app9.png]

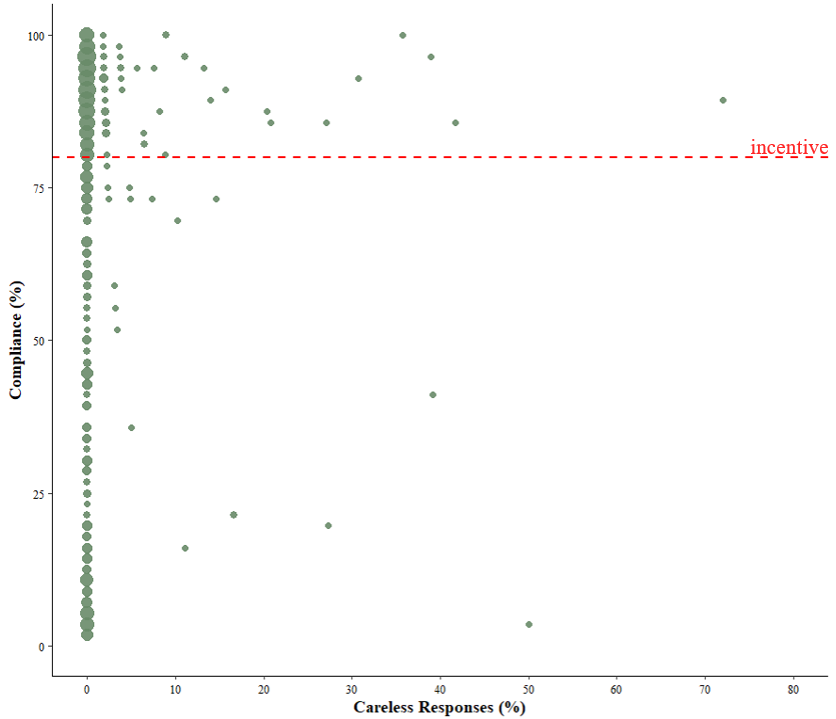

Supplement: Multimedia Appendix 10 [file jmir_v26i1e55712_app10.png]
