# Supplementary material for: Ecological Momentary Assessment of Mental Health Problems Among University Students: Data Quality Evaluation Study
Source: J Med Internet Res. 2024 Dec 10;26:e55712. doi: 10.2196/55712 (PMC11668991; doi:10.2196/55712)
Supplement: Multimedia Appendix 2 [file jmir_v26i1e55712_app2.docx]

| **Item** | **Construct** | **Source** | **Assessment** |
| --- | --- | --- | --- |
| Stressed | Stress | The Experience Samling Method Item Repository^a^ | 4 times a day |
| Tired, little energy | Energy level | PHQ-9, The Experience Samling Method Item Repository^a^ | 4 times a day |
| Having trouble concentrating | Neutral arousal | PHQ-9 | 4 times a day |
| Cheerful, happy | Positive affect, high arousal | The Experience Samling Method Item Repository^a^ | 4 times a day |
| Calm, relaxed | Positive affect, low arousal | SWEMWBS | 4 times a day |
| Optimistic | Positive affect, high arousal | SWEMWBS | 4 times a day |
| Pleasure in doing things | Positive affect, neutral arousal | PHQ-9 | 4 times a day |
| Interest in doing things | Positive affect, neutral arousal | PHQ-9 | 4 times a day |
| Annoyed, irritable | Negative affect, high arousal | GAD-7, The Experience Samling Method Item Repository^a^ | 4 times a day |
| Nervous, anxious, on edge | Negative affect, high arousal | GAD-7, The Experience Samling Method Item Repository^a^ | 4 times a day |
| Worried | Negative affect, high arousal | GAD-7, The Experience Samling Method Item Repository^a^ | 4 times a day |
| Down, depressed | Negative affect, low arousal | PHQ-9, The Experience Samling Method Item Repository^a^ | 4 times a day |
| Montary activity | Montary activity | The Experience Samling Method Item Repository^a^ | 4 times a day |
| Momentary social context | Momentary social context (physical and/or online) | The Experience Samling Method Item Repository^a^ | 4 times a day |
| Momentary physical context | Momentary physical context | The Experience Samling Method Item Repository^a^ | 4 times a day |
| Sleep quantity | Sleep quantity | The Consensus Sleep Diary^b^ | Morning |
| Sleep quality | Sleep quality | Ad hoc | Morning |
| Number of alcoholic drinks consumed the previous day | 24h alcohol consumption | Russell et al.^c^ | Morning |
| Desire to live | Passive suicidal ideation | Salzburg Suicide Questionnaire^d^ | Evening |
| Desire to die | Passive suicidal ideation | Salzburg Suicide Questionnaire^d^ | Evening |
| Thoughts about killing oneself | Active suicidal ideation | Czyz et al.^e^ | Evening |

*^a^ Kirtley, O. J., Hiekkaranta, A. P., Kunkels, Y. K., Eisele, G., Schoefs, S., Kemme, N. D. F., Myin-Germeys, I. (2023, July 13). The Experience Sampling Method (ESM) Item Repository. https://doi.org/10.17605/OSF.IO/KG376*

*^b^ Carney, C. E., Buysse, D. J., Ancoli-Israel, S., Edinger, J. D., Krystal, A. D., Lichstein, K. L., & Morin, C. M. (2012). The consensus sleep diary: standardizing prospective sleep self-monitoring. Sleep, 35(2), 287-302.*

*^c^ Russell, M. A., Almeida, D. M., & Maggs, J. L. (2017). Stressor-related drinking and future alcohol problems among university students. Psychology of Addictive Behaviors, 31(6), 676.*

*^d^ Fartacek, C., Schiepek, G., Kunrath, S., Fartacek, R., & Plöderl, M. (2016). Real-time monitoring of non-linear suicidal dynamics: methodology and a demonstrative case report. Frontiers in psychology, 7, 130.*

*^e^ Czyz, E. K., Horwitz, A. G., Arango, A., & King, C. A. (2019). Short-term change and prediction of suicidal ideation among adolescents: a daily diary study following psychiatric hospitalization. Journal of child psychology and psychiatry, and allied disciplines, 60(7), 732–741.* [*https://doi.org/10.1111/jcpp.12974*](https://doi.org/10.1111/jcpp.12974)
