# Supplementary material for: Ecological Momentary Assessment of Mental Health Problems Among University Students: Data Quality Evaluation Study
Source: J Med Internet Res. 2024 Dec 10;26:e55712. doi: 10.2196/55712 (PMC11668991; doi:10.2196/55712)
Supplement: Multimedia Appendix 3 [file jmir_v26i1e55712_app3.docx]

| **Variables** | **Original instrument** |
| --- | --- |
| Age | Standard question |
| Assigned sex at birth | Williams Institute Best Practices for Asking Questions to Identify Transgender and Other Gender Minority Respondents on Population |
| Type of study | Standard question |
| Primary Nationality | Standard question |
| Role limitations due to physical and emotional health | VR-12 physical component summary |
| Depression | CIDI-SC |
| Anxiety | CIDI-SC |
| Panic attacks | CIDI-SC; Developed by Dr. Kessler based on DSM-5 Panic Disorder diagnostic criteria |
| Manic/Hypomanic episode | CIDI-SC; Developed by Dr. Kessler based on DSM-5 manic/hypomanic episode diagnostic criteria |
| Alcohol use | AUDIT; Developed for WMH-ICS |
| Suicidal ideation | C-SSRS; SITBI; Developed for WMH-ICS |
| Preparatory acts or behaviors | C-SSRS; SITBI; P4 screener |
| Actual attempt | C-SSRS; SITBI |
| Parent education | 2016-2017 Healthy Minds Survey |
| Childhood stressful experiences and physical abuse | CTQ-SF |
| Childhood trauma | CTQ-SF and NSS |
| Bullying | Based on 2017 Youth Risk Behavior Survey |
| Physical and emotional abuse by intimate partner | 2016-2017 Healthy Minds Survey |
| 12-month stressful life events | Based on AAS |

*Acronyms: VR-12 (Veterans RAND 12 Item Health Survey), CIDI-SC (Composite International Diagnostic Interview Screening Scales), WMH-ICS (World Mental Health - International College Surveys), AUDIT (Alcohol Use Disorders Identification Test), ASSIST (Alcohol, Smoking, and Substance Involvement Screening Test use of cannabis, cocaine, other street drugs, prescription drugs), C-SSRS (Columbia-Suicide Severity Rating Scale), SITBI (Self-Injurious Thoughts and Behaviors Interview), CIDI (WHO Composite International Diagnostic Interview), CTQ-SF (Childhood Trauma Questionnaire and the Short Form), AAS (Army STARRS All Army Study Survey), SIS (Supportive and Negative Social Interaction Scale).*
