# Supplementary material for: Ecological Momentary Assessment of Mental Health Problems Among University Students: Data Quality Evaluation Study
Source: J Med Internet Res. 2024 Dec 10;26:e55712. doi: 10.2196/55712 (PMC11668991; doi:10.2196/55712)
Supplement: Multimedia Appendix 4 [file jmir_v26i1e55712_app4.docx]

**Supplementary Table 1.** Correlation coefficients for all items measuring affect using all data from all EMA participants (n= 782).

|  | **Happy** | **Optimistic** | **Interest in doing things** | **Pleasure** | **Relaxed** | **Worried** | **Depressed** | **Upset** | **Nervous** |
| --- | --- | --- | --- | --- | --- | --- | --- | --- | --- |
| **Happy** | 1.00 |  |  |  |  |  |  |  |  |
| **Optimistic** | 0.71 | 1.00 |  |  |  |  |  |  |  |
| **Interest in doing things** | 0.66 | 0.66 | 1.00 |  |  |  |  |  |  |
| **Pleasure** | 0.69 | 0.71 | 0.73 | 1.00 |  |  |  |  |  |
| **Relaxed** | 0.59 | 0.57 | 0.45 | 0.52 | 1.00 |  |  |  |  |
| **Worried** | -0.44 | -0.42 | -0.31 | -0.35 | -0.54 | 1.00 |  |  |  |
| **Depressed** | -0.52 | -0.48 | -0.44 | -0.45 | -0.40 | 0.54 | 1.00 |  |  |
| **Upset** | -0.40 | -0.35 | -0.30 | -0.32 | -0.38 | 0.49 | 0.56 | 1.00 |  |
| **Nervous** | -0.41 | -0.39 | -0.27 | -0.33 | -0.59 | 0.72 | 0.53 | 0.55 | 1.00 |
