# Supplementary material for: Ecological Momentary Assessment of Mental Health Problems Among University Students: Data Quality Evaluation Study
Source: J Med Internet Res. 2024 Dec 10;26:e55712. doi: 10.2196/55712 (PMC11668991; doi:10.2196/55712)
Supplement: Multimedia Appendix 5 [file jmir_v26i1e55712_app5.pdf]

Adapted STROBE Checklist for Reporting EMA Studies (CREMAS)

**Title**		
1. Title	Include ecological momentary assessment in title and key words	See Title and Key Words
**Introduction**		
2. Rationale	Briefly introduce the concept of EMA and provide reasons for utilizing EMA for this study or topic of interests	See Introduction
**Methods**		
3. Training	Indicate if, and by what methods, training of participants for EMA protocol was used	See *Recruitment* subsection of *Methods*
4. Technology	Describe what technology, if any, was used. Include the following information: device (eg, mobile phone, portable computer), model (eg, Nexus 4, iPod), operating system (eg, Android, Windows), and EMA program name	See *Recruitment* subsection of *Methods*
5. Wave Duration	State the number of waves for the study (eg, 2 monitoring periods over the course of 1 year)	See *Study Design and Population* subsection of *Methods*
6. Monitoring period	State the number of days each wave of the study lasted, and how many weekdays versus weekend days	See Study Design and Population subsection of Methods
7. Prompting design	Indicate the prompting strategy used for the study (eg, event-based, interval-based, or a combination of the two). If using interval-based strategy, indicate what type of schedule is used (eg, fixed, random, or hybrid interval)	See *EMA Study Protocol & Measures* subsection of *Methods*
8. Prompt Frequency	Intended frequency of prompts per day. Break down by weekdays and weekend days if applicable	See *EMA Study Protocol & Measures* subsection of *Methods*
9. Design features	Describe any design feature to address potential sources of bias (eg, reactivity) or participant burden (eg, EMA questions appearing in different orders)	This is the central topic of the study. See *Recruitment* subsection of *Methods* for details on selection of participants.
**Results**		
10. Attrition	Indicate participant attrition throughout the study; report attrition rates both by	See *Results*

	monitoring days and waves, if applicable	
11. Prompt delivery	Report number of EMA prompts that were planned to be delivered. If possible, also report the number of EMA prompts that were actually received by participants and indicate reasons for why prompts were not sent out (eg, technical issues or participant noncompliance reason such as phone was powered off)	See *EMA Study Protocol & Measures* subsection of *Methods* and *Results*
12. Latency	Report the amount of time from prompt signal to answering of prompt	See *EMA Study Protocol & Measures* subsection of *Methods*
13. Compliance rate	Report total answered EMA prompts across all subjects and the average number of EMA prompts answered per person. Report compliance rate both by monitoring days and waves, if applicable. Indicate reasons for noncompliance, if known	See *Predictors of EMA Study Participation and Compliance* subsection of *Results*
14. Missing data	Report whether EMA compliance is related to demographic or time-varying variables	See *Predictors of EMA Study Participation and Compliance* subsection of *Results*
**Discussion**		
15. Limitations	Discuss limitations of the study, taking into account sources of potential bias when using EMA methods (eg, reactivity, use of technology)	See *Limitations* subsection of *Discussion*
16. Conclusions	Provide a general interpretation of results and discuss the benefits of using EMA	See *Conclusions* subsection of *Discussion*
