# Supplementary material for: Ecological Momentary Assessment of Mental Health Problems Among University Students: Data Quality Evaluation Study
Source: J Med Internet Res. 2024 Dec 10;26:e55712. doi: 10.2196/55712 (PMC11668991; doi:10.2196/55712)
Supplement: Multimedia Appendix 6 [file jmir_v26i1e55712_app6.docx]

|  | | | Invited to EMA study (n=1259), n (%) | Participation^a^ | | Compliance^b^ | |
| --- | --- | --- | --- | --- | --- | --- | --- |
|  | | |  | Participants (n=782), n (%) | OR^c^ (95% CI)^d^ | Mean % (SD) | Exponential of β (95% CI)^e^ |
|  | | | | | | | |
| Sociodemographic characteristics | | | | | | | |
|  | Sex | | | | | | |
|  |  | Male | 288 (22.9) | 163 (56.6) | Reference | 75.4 (28.2) | Reference |
|  |  | Female | 971 (77.1) | 619 (63.7) | 1.41 (1.06-1.87) | 77.3 (27.5) | 1.02 (0.99-1.05) |
|  | Nationality | | | | | | |
|  |  | Spanish | 1156 (91.8) | 722 (62.5) | Reference | 77.4 (27.3) | Reference |
|  |  | Other | 103 (8.2) | 60 (58.3) | 0.92 (0.61-1.42) | 71.5 (31.2) | 0.94 (0.90-0.98) |
|  | Age (years) | | | | | | |
|  |  | 18-21 | 894 (71) | 577 (64.5) | Reference | 77.1 (27.3) | Reference |
|  |  | 22-25 | 297 (23.6) | 176 (59.3) | 0.80 (0.61-1.06) | 77.9 (28.0) | 1.02 (0.99-1.04) |
|  |  | 26-29 | 36 (2.9) | 21 (58.3) | 0.78 (0.39-1.56) | 67.3 (32.7) | 0.88 (0.82-0.94) |
|  |  | ≥30 | 32 (2.5) | 8 (25.0) | 0.20 (0.08-0.43) | 65.4 (30.6) | 0.86 (0.76-0.96) |
|  | Field of study | | | | | | |
|  |  | Arts and humanities | 154 (12.2) | 88 (57.1) | Reference | 79.2 (26.8) | Reference |
|  |  | Sciences | 141 (11.2) | 94 (66.7) | 1.48 (0.91-2.40) | 77.3 (28.3) | 0.97 (0.93-1.02) |
|  |  | Health sciences | 335 (26.6) | 219 (65.4) | 1.33 (0.89-1.98) | 81.9 (23.6) | 1.03 (0.99-1.07) |
|  |  | Social and legal sciences | 470 (37.3) | 280 (59.6) | 1.08 (0.74-1.57) | 72.8 (30.6) | 0.92 (0.89-0.95) |
|  |  | Engineering and architecture | 159 (12.6) | 101 (63.5) | 1.41 (0.88-2.28) | 75.1 (26.0) | 0.95 (0.91-1.00) |
| Childhood and adolescence adverse experiences (aged <18 years) | | | | | | | |
|  | Parental psychopathology | | | | | | |
|  |  | No | 627 (49.8) | 393 (62.7) | Reference | 77.7 (27.5) | Reference |
|  |  | Yes | 632 (50.2) | 389 (61.6) | 0.98 (0.78-1.25) | 76.1 (27.9) | 0.98 (0.96-1.00) |
|  | Physical abuse | | | | | | |
|  |  | No | 1000 (79.4) | 627 (62.7) | Reference | 77.5 (27.8) | Reference |
|  |  | Yes | 259 (20.6) | 155 (59.8) | 0.93 (0.70-1.25) | 74.5 (27.2) | 0.97 (0.94-0.99) |
|  | Emotional abuse | | | | | | |
|  |  | No | 780 (62) | 501 (64.2) | Reference | 78.1 (26.6) | Reference |
|  |  | Yes | 479 (38) | 281 (58.7) | 0.79 (0.62-1.01) | 74.7 (29.5) | 0.96 (0.94-0.98) |
|  | Sexual abuse | | | | | | |
|  |  | No | 1200 (95.3) | 746 (62.2) | Reference | 77.5 (27.3) | Reference |
|  |  | Yes | 59 (4.7) | 36 (61.0) | 1.04 (0.60-1.85) | 65.4 (32.8) | 0.87 (0.82-0.92) |
|  | Neglect | | | | | | |
|  |  | No | 1060 (84.2) | 661 (62.4) | Reference | 78.0 (27.1) | Reference |
|  |  | Yes | 199 (15.8) | 121 (60.8) | 0.98 (0.71-1.35) | 70.9 (30.1) | 0.92 (0.89-0.94) |
|  | Bully victimization | | | | | | |
|  |  | No | 649 (51.5) | 397 (61.2) | Reference | 78.4 (26.5) | Reference |
|  |  | Yes | 610 (48.5) | 385 (63.1) | 1.14 (0.90-1.44) | 75.3 (28.8) | 0.96 (0.94-0.98) |
|  | Dating violence | | | | | | |
|  |  | No | 1064 (84.5) | 668 (62.8) | Reference | 77.4 (27.4) | Reference |
|  |  | Yes | 195 (15.5) | 114 (58.5) | 0.85 (0.62-1.18) | 74.2 (29.2) | 0.96 (0.93-0.99) |
|  | Number of adverse experiences (age of <18 years) | | | | | | |
|  |  | 0 | 179 (14.2) | 110 (61.5) | Reference | 78.2 (27.4) | Reference |
|  |  | 1 | 338 (26.8) | 222 (65.7) | 1.21 (0.82-1.78) | 77.7 (27.6) | 0.99 (0.95-1.02) |
|  |  | 2 | 273 (21.7) | 172 (63.0) | 1.02 (0.68-1.53) | 78.7 (26.8) | 1.01 (0.97-1.04) |
|  |  | ≥3 | 469 (37.3) | 278 (59.3) | 0.93 (0.64-1.35) | 74.7 (28.4) | 0.96 (0.92-0.99) |
|  | Any adverse experience (age of <18 years) | | | | | | |
|  |  | No | 179 (14.2) | 110 (61.5) | Reference | 78.2 (27.4) | Reference |
|  |  | Yes | 1080 (85.8) | 672 (62.2) | 1.05 (0.75-1.46) | 76.7 (27.7) | 0.98 (0.95-1.01) |
| Recent stressful events (previous 12 months) | | | | | | | |
|  | Life-threatening illness or injury of a friend or family member | | | | | | |
|  |  | No | 750 (59.6) | 458 (61.1) | Reference | 76.7 (28.3) | Reference |
|  |  | Yes | 509 (40.4) | 324 (63.7) | 1.09 (0.86-1.38) | 77.2 (26.9) | 1.00 (0.98-1.03) |
|  | Death of a friend or family member | | | | | | |
|  |  | No | 889 (70.6) | 571 (64.2) | Reference | 76.8 (27.9) | Reference |
|  |  | Yes | 370 (29.4) | 211 (57.0) | 0.73 (0.57-0.94) | 77.1 (27.2) | 1.01 (0.98-1.03) |
|  | Breakup with a romantic partner | | | | | | |
|  |  | No | 919 (73) | 574 (62.5) | Reference | 77.6 (27.4) | Reference |
|  |  | Yes | 340 (27) | 208 (61.2) | 0.92 (0.71-1.20) | 75.1 (28.4) | 0.97 (0.95-0.99) |
|  | Cheating of romantic partner | | | | | | |
|  |  | No | 1186 (94.2) | 734 (61.9) | Reference | 77.4 (27.5) | Reference |
|  |  | Yes | 73 (5.8) | 48 (65.8) | 1.19 (0.72-2.01) | 69.7 (19.6) | 0.90 (0.86-0.94) |
|  | Serious betrayal by someone other than partner | | | | | | |
|  |  | No | 863 (68.5) | 529 (61.3) | Reference | 77.2 (28.0) | Reference |
|  |  | Yes | 396 (31.5) | 253 (63.9) | 1.07 (0.83-1.38) | 76.3 (27.0) | 0.99 (0.96-1.01) |
|  | Serious ongoing arguments or breakup with friends or family members | | | | | | |
|  |  | No | 808 (64.2) | 500 (61.9) | Reference | 76.4 (28.3) | Reference |
|  |  | Yes | 451 (35.8) | 282 (62.5) | 1.04 (0.82-1.33) | 77.9 (26.6) | 1.02 (1.00-1.04) |
|  | Life-threatening illness or injury | | | | | | |
|  |  | No | 1203 (95.6) | 745 (61.9) | Reference | 77.3 (27.2) | Reference |
|  |  | Yes | 56 (4.4) | 37 (66.1) | 1.35 (0.76-2.47) | 68.3 (34.7) | 0.88 (0.84-0.93) |
|  | Serious physical assault | | | | | | |
|  |  | No | 1231 (97.8) | 766 (62.2) | Reference | 76.8 (27.7) | Reference |
|  |  | Yes | 28 (2.2) | 16 (57.1) | 1.01 (0.46-2.26) | 82.9 (24.8) | 1.09 (1.01-1.17) |
|  | Sexual assault or rape | | | | | | |
|  |  | No | 1224 (97.2) | 761 (62.2) | Reference | 77.3 (27.5) | Reference |
|  |  | Yes | 35 (2.8) | 21 (60.0) | 1.02 (0.51-2.15) | 62.7 (30.3) | 0.80 (0.74-0.86) |
|  | Sexual harassment | | | | | | |
|  |  | No | 1043 (82.8) | 641 (61.5) | Reference | 77.5 (27.5) | Reference |
|  |  | Yes | 216 (17.2) | 141 (65.3) | 1.09 (0.80-1.50) | 74.1 (28.4) | 0.95 (0.92-0.97) |
|  | Serious legal issue | | | | | | |
|  |  | No | 1225 (97.3) | 767 (62.6) | Reference | 76.9 (27.7) | Reference |
|  |  | Yes | 34 (2.7) | 15 (44.1) | 0.53 (0.26-1.07) | 74.9 (29.9) | 0.99 (0.91-1.07) |
|  | Number of recent stressful events | | | | | | |
|  |  | 0 | 270 (21.4) | 175 (64.8) | Reference | 74.9 (30.8) | Reference |
|  |  | 1 | 274 (21.8) | 162 (59.1) | 0.80 (0.56-1.14) | 81.1 (23.8) | 1.09 (1.05-1.13) |
|  |  | 2 | 294 (23.4) | 185 (62.9) | 0.92 (0.65-1.31) | 76.6 (28.0) | 1.02 (0.99-1.06) |
|  |  | ≥3 | 421 (33.4) | 260 (61.8) | 0.82 (0.59-1.15) | 75.9 (27.4) | 1.01 (0.98-1.04) |
|  | Any recent stressful event | | | | | | |
|  |  | No | 270 (21.4) | 175 (64.8) | Reference | 74.9 (30.9) | Reference |
|  |  | Yes | 989 (78.6) | 607 (61.4) | 0.85 (0.64-1.13) | 77.5 (26.7) | 1.03 (1.01-1.06) |
| Mental disorders in the previous 12 months | | | | | | | |
|  | Major depressive disorder | | | | | | |
|  |  | No | 729 (57.9) | 465 (63.8) | Reference | 78.3 (27.1) | Reference |
|  |  | Yes | 530 (42.1) | 317 (59.8) | 0.82 (0.64-1.04) | 74.9 (28.4) | 0.95 (0.93-0.97) |
|  | Generalized anxiety disorder | | | | | | |
|  |  | No | 963 (76.5) | 602 (62.5) | Reference | 77.9 (26.4) | Reference |
|  |  | Yes | 296 (23.5) | 180 (60.8) | 0.95 (0.72-1.25) | 73.7 (31.6) | 0.95 (0.92-0.97) |
|  | Alcohol use disorder | | | | | | |
|  |  | No | 1188 (94.4) | 736 (62.0) | Reference | 77.4 (27.4) | Reference |
|  |  | Yes | 71 (5.6) | 46 (64.8) | 1.11 (0.67-1.87) | 68.6 (30.5) | 0.89 (0.85-0.93) |
|  | Suicidal ideation | | | | | | |
|  |  | No | 862 (68.5) | 540 (62.6) | Reference | 77.5 (27.4) | Reference |
|  |  | Yes | 397 (31.5) | 242 (61) | 0.92 (0.72-1.19) | 75.6 (28.3) | 0.97 (0.95-0.99) |
|  | Suicide plan | | | | | | |
|  |  | No | 1057 (84) | 667 (63.1) | Reference | 77.0 (27.7) | Reference |
|  |  | Yes | 202 (16) | 115 (56.9) | 0.78 (0.57-1.07) | 76.3 (27.5) | 0.99 (0.96-1.02) |
|  | Suicide attempt | | | | | | |
|  |  | No | 1238 (98.3) | 775 (62.6) | Reference | 76.9 (27.8) | Reference |
|  |  | Yes | 21 (1.7) | 7 (33.3) | 0.26 (0.10-0.64) | 81.9 (17.2) | 1.05 (0.94-1.17) |

^a^Defined as those who completed at least one momentary assessment (n=782) of those invited to participate in the EMA study (N=1259).

^b^Defined as the percentage of momentary assessments completed per individual divided by the maximum number of momentary assessments allowed by the design per individual (ie, 56) among EMA study participants (n=782).

^c^OR: odds ratio.

^d^Each row represents a separate logistic regression model, each time adjusting for sociodemographic and college-related variables (ie, sex, nationality, age, and field of study).

^e^Each row represents a separate Poisson regression model, each time adjusting for sociodemographic and college-related variables (ie, sex, nationality, age, and field of study).
