# Supplementary material for: Ecological Momentary Assessment of Mental Health Problems Among University Students: Data Quality Evaluation Study
Source: J Med Internet Res. 2024 Dec 10;26:e55712. doi: 10.2196/55712 (PMC11668991; doi:10.2196/55712)
Supplement: Multimedia Appendix 8 [file jmir_v26i1e55712_app8.docx]

**Supplementary Table 2.** Within-person reliability scores for positive (PA) and negative (NA) affect, compliance and percentage of answered assessments responded carelessly for all EMA participants (n= 782).

| **ID** | **Raw Alpha (PA) [1]** | **Raw Alpha (NA) [1]** | **Compliance [2]** | **Assessments with Careless Responding (%) [3]** |
| --- | --- | --- | --- | --- |
| anxdep_666 | 0.85 | 0.69 | 100 | 0 |
| anxdep_199 | 0.91 | 0.3 | 100 | 0 |
| cont_245 | 0.87 | 0.86 | 100 | 0 |
| anxdep_622 | 0.12 | 0.09 | 100 | 0 |
| anxdep_621 | 0.62 | 0.83 | 100 | 35.7 |
| anxdep_116 | 0.86 | 0.65 | 100 | 0 |
| anxdep_10 | 0.6 | 0.73 | 100 | 0 |
| anxdep_577 | 0.94 | 0.87 | 100 | 0 |
| anxdep_180 | 0.47 | 0.35 | 100 | 0 |
| sui_744 | 0.84 | 0.53 | 100 | 0 |
| anxdep_333 | 0.82 | 0.59 | 100 | 0 |
| alc_526 | 0.88 | 0.86 | 100 | 0 |
| cont_570 | 0.81 | 0.67 | 100 | 0 |
| alc_358 | 0.79 | 0.62 | 100 | 0 |
| sui_452 | 0.78 | 0.45 | 100 | 0 |
| sui_506 | 0.86 | 0.88 | 100 | 1.8 |
| sui_453 | 0.84 | 0.78 | 100 | 8.9 |
| sui_47 | 0.76 | 0.83 | 100 | 0 |
| cont_619 | 0.95 | 0.91 | 100 | 0 |
| anxdep_411 | 0.89 | 0.86 | 100 | 0 |
| cont_187 | 0.79 | 0.5 | 100 | 0 |
| sui_104 | 0.86 | 0.67 | 100 | 0 |
| sui_35 | 0.75 | 0.76 | 100 | 8.9 |
| alc_715 | 0.84 | 0.67 | 98.2 | 0 |
| cont_84 | 0.72 | 0.55 | 98.2 | 0 |
| anxdep_302 | 0.9 | 0.84 | 98.2 | 3.6 |
| alc_345 | 0.73 | 0.6 | 98.2 | 0 |
| sui_455 | 0.78 | 0.78 | 98.2 | 0 |
| anxdep_616 | 0.65 | 0.89 | 98.2 | 0 |
| anxdep_54 | 0.92 | 0.63 | 98.2 | 0 |
| alc_159 | 0.8 | 0.45 | 98.2 | 0 |
| anxdep_636 | 0.71 | 0.21 | 98.2 | 0 |
| anxdep_129 | 0.91 | 0.93 | 98.2 | 0 |
| anxdep_429 | 0.91 | 0.79 | 98.2 | 0 |
| anxdep_122 | 0.84 | 0.65 | 98.2 | 0 |
| alc_470 | 0.7 | 0.94 | 98.2 | 0 |
| anxdep_678 | 0.39 | 0.64 | 98.2 | 0 |
| sui_686 | 0.81 | 0.74 | 98.2 | 0 |
| alc_628 | 0.7 | 0.51 | 98.2 | 0 |
| anxdep_191 | 0.68 | 0.67 | 98.2 | 0 |
| sui_750 | 0.87 | 0.63 | 98.2 | 0 |
| anxdep_207 | 0.82 | 0.62 | 98.2 | 0 |
| sui_749 | 0.79 | 0.75 | 98.2 | 0 |
| anxdep_707 | 0.81 | 0.91 | 98.2 | 0 |
| cont_494 | 0.9 | 0.46 | 98.2 | 0 |
| sui_532 | 0.8 | 0.44 | 98.2 | 0 |
| anxdep_478 | 0.77 | 0.79 | 98.2 | 0 |
| anxdep_523 | 0.75 | 0.57 | 98.2 | 0 |
| sui_404 | 0.91 | 0.78 | 98.2 | 0 |
| anxdep_102 | 0.82 | 0.83 | 98.2 | 1.8 |
| sui_105 | 0.73 | 0.61 | 98.2 | 0 |
| alc_573 | 0.83 | 0.68 | 98.2 | 0 |
| anxdep_193 | 0.82 | 0.69 | 98.2 | 0 |
| anxdep_629 | 0.69 | 0.8 | 98.2 | 0 |
| cont_496 | 0.84 | 0.59 | 98.2 | 0 |
| sui_58 | 0.9 | 0.83 | 98.2 | 0 |
| alc_88 | 0.75 | 0.38 | 98.2 | 0 |
| anxdep_740 | 0.79 | 0.44 | 98.2 | 0 |
| cont_359 | 0.92 | -0.04 | 98.2 | 0 |
| cont_655 | 0.85 | 0.9 | 98.2 | 0 |
| alc_182 | 0.62 | 0.59 | 98.2 | 0 |
| anxdep_681 | 0.51 | 0.73 | 98.2 | 0 |
| cont_576 | 0.6 | 0.47 | 98.2 | 0 |
| sui_433 | 0.91 | 0.9 | 98.2 | 0 |
| anxdep_70 | 0.9 | 0.86 | 98.2 | 0 |
| cont_152 | 0.79 | 0.76 | 98.2 | 0 |
| cont_413 | 0.68 | 0.9 | 98.2 | 0 |
| anxdep_301 | 0.84 | 0.78 | 98.2 | 0 |
| sui_415 | 0.62 | 0.8 | 98.2 | 0 |
| cont_63 | 0.87 | 0.59 | 98.2 | 0 |
| anxdep_571 | 0.9 | 0.68 | 96.4 | 0 |
| anxdep_492 | 0.58 | 0.59 | 96.4 | 0 |
| cont_261 | 0.36 | 0.73 | 96.4 | 38.9 |
| cont_335 | 0.57 | 0.59 | 96.4 | 0 |
| alc_282 | 0.8 | 0.49 | 96.4 | 0 |
| alc_76 | 0.6 | 0.2 | 96.4 | 0 |
| alc_593 | 0.78 | 0.71 | 96.4 | 0 |
| sui_340 | 0.94 | 0.77 | 96.4 | 0 |
| alc_495 | 0.86 | 0.81 | 96.4 | 0 |
| sui_265 | 0.83 | 0.78 | 96.4 | 0 |
| sui_123 | 0.84 | 0.8 | 96.4 | 0 |
| cont_108 | 0.7 | 0.67 | 96.4 | 0 |
| anxdep_533 | 0.06 | 0.74 | 96.4 | 1.9 |
| anxdep_500 | 0.95 | 0.81 | 96.4 | 0 |
| anxdep_313 | 0.22 | 0.31 | 96.4 | 0 |
| anxdep_388 | 0.89 | 0.7 | 96.4 | 0 |
| anxdep_482 | 0.81 | 0.6 | 96.4 | 0 |
| cont_212 | 0.89 | 0.79 | 96.4 | 0 |
| alc_692 | 0.54 | -0.41 | 96.4 | 0 |
| anxdep_615 | 0.86 | 0.86 | 96.4 | 0 |
| alc_677 | 0.88 | 0.9 | 96.4 | 0 |
| sui_27 | 0.87 | 0.66 | 96.4 | 0 |
| sui_164 | 0.9 | 0.72 | 96.4 | 0 |
| sui_634 | 0.81 | 0.55 | 96.4 | 0 |
| sui_339 | 0.88 | 0.72 | 96.4 | 0 |
| anxdep_90 | 0.77 | 0.74 | 96.4 | 0 |
| sui_184 | 0.75 | 0.78 | 96.4 | 0 |
| alc_259 | 0.82 | 0.78 | 96.4 | 11.1 |
| alc_179 | 0.56 | 0.73 | 96.4 | 0 |
| cont_317 | 0.7 | 0.33 | 96.4 | 0 |
| sui_738 | 0.87 | 0.73 | 96.4 | 0 |
| alc_145 | 0.64 | 0.65 | 96.4 | 0 |
| cont_700 | 0.74 | 0.68 | 96.4 | 0 |
| cont_741 | 0.8 | 0.37 | 96.4 | 0 |
| cont_612 | 0.09 | 0.28 | 96.4 | 0 |
| cont_683 | 0.86 | 0.71 | 96.4 | 0 |
| alc_539 | 0.9 | 0.75 | 96.4 | 0 |
| alc_242 | 0.71 | 0.64 | 96.4 | 0 |
| cont_303 | 0.88 | 0.29 | 96.4 | 0 |
| cont_394 | 0.83 | 0.42 | 96.4 | 0 |
| alc_57 | 0.87 | 0.81 | 96.4 | 0 |
| cont_472 | 0.84 | 0.39 | 96.4 | 0 |
| sui_254 | 0.88 | 0.68 | 96.4 | 0 |
| sui_263 | 0.86 | 0.71 | 96.4 | 0 |
| cont_6 | 0.43 | 0.19 | 96.4 | 0 |
| cont_503 | 0.52 | 0 | 96.4 | 0 |
| sui_375 | 0.69 | 0.69 | 96.4 | 0 |
| cont_236 | 0.66 | 0.72 | 96.4 | 0 |
| alc_316 | 0.74 | 0.01 | 96.4 | 0 |
| alc_364 | 0.82 | 0.66 | 96.4 | 0 |
| anxdep_558 | 0.85 | 0.89 | 96.4 | 11.1 |
| sui_684 | 0.86 | 0.9 | 96.4 | 0 |
| cont_99 | 0.71 | 0.88 | 96.4 | 0 |
| alc_5 | 0.67 | 0.35 | 96.4 | 0 |
| anxdep_52 | 0.85 | 0.75 | 96.4 | 0 |
| cont_579 | 0.82 | 0.72 | 96.4 | 0 |
| alc_306 | 0.75 | 0.69 | 96.4 | 0 |
| sui_235 | 0.89 | 0.84 | 96.4 | 0 |
| sui_238 | 0.71 | 0.66 | 96.4 | 0 |
| alc_60 | 0.92 | 0.83 | 96.4 | 0 |
| anxdep_190 | 0.81 | 0.6 | 96.4 | 0 |
| anxdep_251 | 0.7 | 0.73 | 96.4 | 0 |
| cont_460 | 0.81 | 0.47 | 96.4 | 3.7 |
| cont_181 | 0.77 | 0.45 | 96.4 | 0 |
| anxdep_711 | 0.41 | 0.65 | 96.4 | 0 |
| sui_782 | 0.89 | 0.67 | 96.4 | 0 |
| cont_255 | 0.34 | -0.04 | 96.4 | 0 |
| cont_667 | 0.86 | 0.73 | 96.4 | 1.9 |
| cont_626 | 0.65 | 0.57 | 96.4 | 0 |
| cont_521 | 0.33 | 0.71 | 96.4 | 0 |
| anxdep_111 | 0.86 | 0.71 | 96.4 | 0 |
| anxdep_737 | 0.91 | 0.88 | 96.4 | 0 |
| cont_201 | 0.63 | 0.68 | 96.4 | 0 |
| sui_578 | 0.69 | 0.57 | 96.4 | 0 |
| sui_138 | 0.89 | 0.8 | 96.4 | 0 |
| alc_447 | 0.82 | 0.7 | 94.6 | 7.5 |
| cont_197 | 0.69 | 0.7 | 94.6 | 0 |
| sui_143 | 0.78 | 0.62 | 94.6 | 3.8 |
| sui_660 | 0.89 | 0.84 | 94.6 | 0 |
| cont_774 | 0.89 | 0.38 | 94.6 | 0 |
| cont_342 | 0.69 | 0.54 | 94.6 | 0 |
| alc_671 | 0.7 | 0.41 | 94.6 | 0 |
| anxdep_293 | 0.88 | 0.83 | 94.6 | 0 |
| alc_213 | 0.87 | 0.83 | 94.6 | 3.8 |
| anxdep_390 | 0.7 | 0.7 | 94.6 | 0 |
| alc_659 | 0.69 | 0.91 | 94.6 | 0 |
| alc_281 | 0.59 | 0.68 | 94.6 | 0 |
| sui_45 | 0.88 | 0.84 | 94.6 | 0 |
| alc_67 | 0.18 | 0.27 | 94.6 | 0 |
| alc_324 | 0.75 | 0.81 | 94.6 | 0 |
| alc_705 | 0.75 | 0.8 | 94.6 | 0 |
| alc_202 | 0.73 | 0.87 | 94.6 | 0 |
| alc_189 | 0.71 | 0.74 | 94.6 | 0 |
| anxdep_147 | 0.65 | 0.63 | 94.6 | 0 |
| sui_703 | 0.78 | 0.77 | 94.6 | 0 |
| alc_426 | 0.88 | 0.82 | 94.6 | 0 |
| anxdep_18 | 0.7 | 0.43 | 94.6 | 0 |
| cont_370 | 0.8 | 0.67 | 94.6 | 0 |
| cont_253 | 0.92 | 0.74 | 94.6 | 0 |
| cont_14 | 0.68 | 0.75 | 94.6 | 0 |
| alc_529 | 0.38 | 0.48 | 94.6 | 13.2 |
| anxdep_589 | 0.91 | 0.71 | 94.6 | 0 |
| alc_295 | 0.71 | 0.73 | 94.6 | 0 |
| cont_127 | 0.8 | 0.62 | 94.6 | 0 |
| sui_174 | 0.85 | 0.79 | 94.6 | 0 |
| anxdep_16 | 0.67 | 0.1 | 94.6 | 0 |
| anxdep_56 | 0.91 | 0.72 | 94.6 | 0 |
| alc_160 | 0.65 | 0.56 | 94.6 | 0 |
| alc_183 | 0.92 | 0.79 | 94.6 | 0 |
| sui_305 | 0.8 | 0.89 | 94.6 | 0 |
| sui_172 | 0.87 | 0.63 | 94.6 | 0 |
| alc_257 | 0.84 | 0.82 | 94.6 | 0 |
| anxdep_383 | 0.84 | 0.85 | 94.6 | 0 |
| sui_731 | 0.92 | 0.73 | 94.6 | 0 |
| cont_120 | 0.75 | 0.76 | 94.6 | 0 |
| cont_620 | 0.76 | 0.76 | 94.6 | 0 |
| anxdep_214 | 0.5 | 0.44 | 94.6 | 0 |
| cont_114 | 0.78 | 0.76 | 94.6 | 0 |
| cont_520 | 0.74 | 0.3 | 94.6 | 0 |
| sui_587 | 0.73 | 0.64 | 94.6 | 0 |
| cont_195 | 0.64 | 0.51 | 94.6 | 0 |
| alc_443 | 0.8 | 0.86 | 94.6 | 5.7 |
| sui_307 | 0.79 | 0.72 | 94.6 | 0 |
| alc_430 | 0.86 | 0.86 | 94.6 | 0 |
| alc_694 | 0.84 | 0.57 | 94.6 | 0 |
| anxdep_490 | 0.81 | 0.64 | 94.6 | 0 |
| cont_781 | 0.85 | 0.71 | 94.6 | 1.9 |
| alc_489 | 0.79 | 0.79 | 94.6 | 0 |
| anxdep_96 | 0.61 | 0.7 | 94.6 | 0 |
| alc_270 | 0.77 | 0.71 | 94.6 | 0 |
| anxdep_702 | 0.85 | 0.74 | 94.6 | 0 |
| cont_400 | 0.75 | 0.65 | 94.6 | 0 |
| cont_275 | 0.81 | 0.74 | 94.6 | 0 |
| anxdep_59 | 0.75 | 0.72 | 94.6 | 0 |
| cont_55 | 0.85 | 0.63 | 94.6 | 0 |
| cont_735 | 0.73 | 0.88 | 94.6 | 0 |
| sui_118 | 0.64 | 0.43 | 94.6 | 0 |
| anxdep_332 | 0.88 | 0.72 | 94.6 | 1.9 |
| anxdep_733 | 0.75 | 0.89 | 94.6 | 0 |
| sui_260 | 0.59 | 0.47 | 94.6 | 0 |
| cont_624 | 0.74 | 0.59 | 94.6 | 0 |
| anxdep_657 | 0.83 | 0.7 | 94.6 | 0 |
| anxdep_24 | 0.76 | 0.53 | 94.6 | 0 |
| anxdep_535 | 0.79 | 0.64 | 94.6 | 0 |
| anxdep_151 | 0.84 | 0.7 | 94.6 | 0 |
| cont_687 | 0.88 | 0.61 | 94.6 | 0 |
| sui_229 | 0.85 | 0.85 | 92.9 | 0 |
| alc_150 | 0.88 | 0.82 | 92.9 | 0 |
| sui_83 | 0.9 | 0.71 | 92.9 | 1.9 |
| anxdep_132 | 0.86 | 0.79 | 92.9 | 0 |
| cont_674 | 0.65 | 0.5 | 92.9 | 1.9 |
| anxdep_742 | 0.85 | 0.71 | 92.9 | 0 |
| anxdep_484 | 0.76 | 0.47 | 92.9 | 0 |
| anxdep_277 | 0.77 | 0.63 | 92.9 | 0 |
| cont_459 | 0.73 | 0.75 | 92.9 | 0 |
| anxdep_26 | 0.93 | 0.6 | 92.9 | 0 |
| anxdep_759 | 0.77 | 0.6 | 92.9 | 0 |
| alc_670 | 0.57 | 0.66 | 92.9 | 0 |
| sui_695 | 0.86 | 0.66 | 92.9 | 3.8 |
| sui_691 | 0.87 | 0.67 | 92.9 | 30.8 |
| alc_373 | -0.21 | 0.5 | 92.9 | 0 |
| alc_441 | 0.71 | 0.67 | 92.9 | 0 |
| anxdep_299 | 0.8 | 0.83 | 92.9 | 0 |
| alc_258 | 0.89 | 0.73 | 92.9 | 0 |
| cont_491 | 0.84 | 0.43 | 92.9 | 0 |
| anxdep_81 | 0.86 | 0.65 | 92.9 | 0 |
| alc_343 | 0.72 | 0.52 | 92.9 | 0 |
| alc_44 | 0.91 | 0.58 | 92.9 | 0 |
| anxdep_746 | 0.86 | 0.9 | 92.9 | 0 |
| alc_13 | 0.71 | 0.51 | 92.9 | 0 |
| cont_177 | 0.33 | 0.65 | 92.9 | 0 |
| cont_424 | 0.88 | 0.75 | 92.9 | 1.9 |
| sui_501 | 0.85 | 0.77 | 92.9 | 0 |
| sui_608 | 0.77 | 0.74 | 92.9 | 0 |
| anxdep_714 | 0.78 | 0.75 | 92.9 | 0 |
| sui_157 | 0.78 | 0.85 | 92.9 | 0 |
| sui_456 | 0.88 | 0.69 | 92.9 | 0 |
| anxdep_712 | 0.65 | 0.56 | 92.9 | 0 |
| cont_770 | 0.57 | 0.63 | 92.9 | 0 |
| sui_283 | 0.81 | 0.58 | 92.9 | 0 |
| anxdep_326 | 0.86 | 0.77 | 92.9 | 0 |
| cont_50 | 0.3 | 0.19 | 92.9 | 0 |
| anxdep_334 | 0.83 | 0.75 | 92.9 | 0 |
| alc_39 | 0.87 | 0.83 | 92.9 | 0 |
| cont_162 | 0.76 | 0.5 | 92.9 | 0 |
| cont_458 | 0.88 | 0.65 | 92.9 | 0 |
| alc_776 | 0.8 | 0.59 | 92.9 | 0 |
| cont_717 | 0.24 | #N/A | 92.9 | 0 |
| sui_599 | 0.87 | 0.69 | 92.9 | 0 |
| alc_631 | 0.41 | 0.51 | 92.9 | 0 |
| anxdep_457 | 0.76 | 0.42 | 92.9 | 0 |
| anxdep_284 | 0.91 | 0.87 | 92.9 | 0 |
| cont_517 | 0.82 | 0.74 | 92.9 | 0 |
| cont_464 | 0.87 | 0.73 | 92.9 | 0 |
| alc_219 | 0.64 | 0.72 | 92.9 | 0 |
| alc_516 | 0.93 | 0.82 | 92.9 | 0 |
| cont_672 | 0.84 | 0.12 | 92.9 | 0 |
| cont_574 | 0.72 | 0.23 | 92.9 | 0 |
| sui_119 | 0.89 | 0.8 | 92.9 | 1.9 |
| anxdep_497 | 0.81 | 0.59 | 92.9 | 0 |
| cont_348 | 0.63 | 0.61 | 92.9 | 0 |
| cont_395 | 0.87 | 0.53 | 92.9 | 0 |
| anxdep_228 | 0.73 | 0.77 | 92.9 | 0 |
| sui_721 | 0.56 | 0.63 | 92.9 | 0 |
| anxdep_95 | 0.95 | 0.97 | 92.9 | 0 |
| sui_79 | 0.46 | 0.36 | 92.9 | 0 |
| anxdep_728 | 0.78 | 0.93 | 92.9 | 0 |
| sui_113 | 0.8 | 0.83 | 92.9 | 0 |
| anxdep_505 | 0.84 | 0.71 | 92.9 | 0 |
| anxdep_144 | 0.77 | 0.48 | 92.9 | 0 |
| cont_266 | 0.86 | 0.61 | 92.9 | 0 |
| alc_698 | 0.83 | 0.19 | 91.1 | 0 |
| alc_450 | 0.8 | 0.9 | 91.1 | 0 |
| alc_250 | 0.84 | 0.51 | 91.1 | 0 |
| cont_422 | 0.92 | 0.88 | 91.1 | 0 |
| cont_256 | 0.72 | 0.86 | 91.1 | 0 |
| cont_161 | 0.8 | 0.5 | 91.1 | 0 |
| anxdep_203 | 0.88 | 0.94 | 91.1 | 0 |
| sui_136 | 0.93 | 0.93 | 91.1 | 3.9 |
| cont_246 | 0.86 | 0.66 | 91.1 | 0 |
| anxdep_361 | 0.79 | 0.87 | 91.1 | 0 |
| anxdep_344 | 0.86 | 0.77 | 91.1 | 0 |
| alc_158 | 0.87 | 0.7 | 91.1 | 0 |
| sui_772 | 0.75 | 0.76 | 91.1 | 0 |
| anxdep_220 | 0.76 | 0.62 | 91.1 | 0 |
| alc_290 | 0.34 | 0.8 | 91.1 | 0 |
| alc_208 | 0.88 | 0.94 | 91.1 | 0 |
| alc_409 | 0.84 | 0.66 | 91.1 | 0 |
| cont_690 | 0.81 | 0.57 | 91.1 | 0 |
| alc_392 | 0.87 | 0.72 | 91.1 | 0 |
| sui_594 | 0.61 | 0.79 | 91.1 | 15.7 |
| sui_217 | 0.81 | 0.44 | 91.1 | 0 |
| anxdep_221 | 0.83 | 0.7 | 91.1 | 0 |
| sui_725 | 0.78 | 0.55 | 91.1 | 0 |
| alc_337 | 0.8 | 0.69 | 91.1 | 0 |
| alc_320 | 0.88 | 0.83 | 91.1 | 0 |
| alc_85 | 0.85 | 0.65 | 91.1 | 0 |
| cont_222 | 0.83 | 0.66 | 91.1 | 0 |
| alc_298 | 0.59 | 0.57 | 91.1 | 0 |
| alc_567 | 0.77 | 0.62 | 91.1 | 0 |
| alc_485 | 0.84 | 0.66 | 91.1 | 0 |
| anxdep_748 | 0.79 | 0.84 | 91.1 | 0 |
| alc_80 | 0.44 | 0.06 | 91.1 | 0 |
| alc_272 | 0.6 | 0.7 | 91.1 | 0 |
| anxdep_668 | 0.87 | 0.87 | 91.1 | 0 |
| cont_680 | 0.7 | 0.61 | 91.1 | 0 |
| sui_575 | 0.83 | 0.8 | 91.1 | 2 |
| sui_662 | 0.89 | 0.69 | 91.1 | 0 |
| cont_739 | 0.83 | 0.91 | 91.1 | 2 |
| anxdep_48 | 0.78 | 0.74 | 91.1 | 0 |
| anxdep_713 | 0.86 | 0.59 | 91.1 | 0 |
| alc_437 | 0.87 | 0.78 | 91.1 | 0 |
| alc_598 | 0.75 | 0.46 | 91.1 | 0 |
| sui_131 | 0.8 | 0.66 | 91.1 | 0 |
| anxdep_423 | 0.75 | 0.66 | 91.1 | 0 |
| alc_139 | 0.88 | 0.81 | 91.1 | 0 |
| cont_524 | 0.82 | 0.26 | 91.1 | 0 |
| anxdep_682 | 0.63 | 0.52 | 91.1 | 0 |
| alc_479 | 0.92 | 0.81 | 91.1 | 0 |
| sui_397 | 0.91 | 0.69 | 91.1 | 0 |
| cont_216 | 0.94 | 0.66 | 91.1 | 0 |
| cont_269 | 0.79 | 0.06 | 91.1 | 0 |
| alc_736 | 0.76 | 0.46 | 91.1 | 0 |
| sui_646 | 0.85 | 0.79 | 91.1 | 0 |
| alc_488 | 0.65 | 0.32 | 91.1 | 0 |
| sui_586 | 0.89 | 0.88 | 91.1 | 0 |
| alc_554 | 0.64 | 0.51 | 91.1 | 0 |
| anxdep_372 | 0.8 | 0.66 | 91.1 | 0 |
| alc_169 | 0.76 | 0.58 | 91.1 | 0 |
| cont_367 | 0.86 | 0.56 | 91.1 | 0 |
| cont_380 | 0.76 | 0.81 | 91.1 | 0 |
| alc_73 | 0.75 | 0.55 | 91.1 | 0 |
| alc_205 | 0.59 | 0.66 | 91.1 | 0 |
| anxdep_8 | 0.86 | 0.67 | 91.1 | 0 |
| alc_300 | 0.82 | 0.56 | 91.1 | 0 |
| sui_734 | 0.94 | 0.97 | 91.1 | 0 |
| alc_267 | 0.46 | 0.43 | 91.1 | 0 |
| cont_130 | 0.67 | 0.89 | 91.1 | 0 |
| anxdep_755 | 0.73 | 0.74 | 91.1 | 0 |
| cont_124 | 0.72 | 0.26 | 91.1 | 0 |
| sui_355 | 0.84 | 0.73 | 91.1 | 0 |
| alc_89 | 0.38 | 0.68 | 89.3 | 0 |
| sui_22 | 0.73 | 0.78 | 89.3 | 0 |
| sui_51 | 0.87 | 0.79 | 89.3 | 0 |
| anxdep_654 | 0.77 | 0.81 | 89.3 | 0 |
| alc_540 | 0.81 | 0.72 | 89.3 | 0 |
| anxdep_556 | 0.92 | 0.89 | 89.3 | 0 |
| alc_592 | 0.89 | 0.64 | 89.3 | 0 |
| cont_527 | 0.66 | 0.5 | 89.3 | 0 |
| alc_428 | 0.8 | 0.45 | 89.3 | 0 |
| cont_679 | 0.92 | 0.9 | 89.3 | 0 |
| cont_280 | 0.67 | 0.54 | 89.3 | 0 |
| anxdep_729 | 0.82 | 0.72 | 89.3 | 0 |
| alc_21 | 0.77 | 0.45 | 89.3 | 0 |
| sui_727 | 0.88 | 0.87 | 89.3 | 0 |
| alc_568 | 0.76 | 0.46 | 89.3 | 0 |
| alc_462 | 0.72 | 0.52 | 89.3 | 0 |
| alc_617 | 0.68 | 0.67 | 89.3 | 0 |
| alc_706 | 0.52 | 0.57 | 89.3 | 0 |
| anxdep_210 | 0.75 | 0.64 | 89.3 | 0 |
| alc_605 | 0.39 | -0.22 | 89.3 | 0 |
| alc_669 | 0.82 | 0.68 | 89.3 | 0 |
| alc_223 | 0.68 | 0.52 | 89.3 | 0 |
| sui_91 | 0.72 | 0.62 | 89.3 | 0 |
| alc_66 | 0.4 | -0.22 | 89.3 | 14 |
| cont_653 | 0.62 | 0.68 | 89.3 | 0 |
| anxdep_664 | 0.89 | 0.76 | 89.3 | 0 |
| anxdep_473 | 0.9 | 0.78 | 89.3 | 0 |
| alc_596 | 0.78 | 0.31 | 89.3 | 0 |
| cont_588 | 0.39 | 0.3 | 89.3 | 0 |
| sui_308 | 0.82 | 0.64 | 89.3 | 0 |
| alc_572 | 0.86 | 0.83 | 89.3 | 0 |
| alc_753 | 0.73 | 0.56 | 89.3 | 0 |
| alc_232 | 0.85 | 0.75 | 89.3 | 0 |
| anxdep_336 | 0.87 | 0.74 | 89.3 | 0 |
| alc_552 | 0.75 | 0.83 | 89.3 | 0 |
| cont_349 | 0.66 | 0.34 | 89.3 | 0 |
| alc_43 | 0.81 | 0.5 | 89.3 | 2 |
| cont_585 | 0.88 | 0.64 | 89.3 | 0 |
| anxdep_2 | 0.86 | 0.92 | 89.3 | 72 |
| cont_68 | 0.81 | 0.45 | 89.3 | 0 |
| anxdep_92 | 0.58 | 0.39 | 89.3 | 0 |
| anxdep_547 | 0.75 | 0.48 | 89.3 | 0 |
| cont_31 | 0.63 | 0.6 | 89.3 | 0 |
| anxdep_519 | 0.92 | 0.93 | 89.3 | 0 |
| alc_134 | 0.67 | 0.36 | 89.3 | 0 |
| anxdep_230 | 0.72 | 0.38 | 89.3 | 0 |
| sui_37 | 0.95 | 0.94 | 89.3 | 0 |
| anxdep_652 | 0.79 | 0.66 | 89.3 | 0 |
| anxdep_1 | 0.86 | 0.84 | 89.3 | 0 |
| sui_40 | 0.92 | 0.39 | 89.3 | 0 |
| anxdep_708 | 0.88 | 0.88 | 87.5 | 2 |
| sui_559 | 0.85 | 0.75 | 87.5 | 0 |
| cont_449 | 0.95 | 0.8 | 87.5 | 0 |
| alc_633 | 0.87 | 0.75 | 87.5 | 0 |
| anxdep_241 | 0.84 | 0.34 | 87.5 | 0 |
| sui_360 | 0.91 | 0.85 | 87.5 | 0 |
| sui_553 | 0.79 | 0.64 | 87.5 | 0 |
| anxdep_757 | 0.63 | 0.73 | 87.5 | 0 |
| alc_699 | 0.92 | 0.8 | 87.5 | 0 |
| alc_606 | 0.84 | 0.8 | 87.5 | 0 |
| alc_651 | 0.8 | 0.69 | 87.5 | 0 |
| alc_614 | 0.77 | 0.66 | 87.5 | 0 |
| alc_176 | 0.85 | 0.82 | 87.5 | 0 |
| cont_483 | 0.41 | 0.03 | 87.5 | 0 |
| cont_767 | 0.8 | 0.74 | 87.5 | 0 |
| sui_405 | 0.87 | 0.86 | 87.5 | 8.2 |
| cont_94 | 0.77 | 0.63 | 87.5 | 0 |
| alc_142 | 0.91 | 0.91 | 87.5 | 0 |
| anxdep_454 | 0.72 | 0.77 | 87.5 | 0 |
| cont_329 | 0.45 | 0.63 | 87.5 | 0 |
| cont_630 | 0.76 | 0.46 | 87.5 | 0 |
| alc_611 | 0.7 | 0.77 | 87.5 | 0 |
| sui_209 | 0.76 | 0.71 | 87.5 | 0 |
| alc_377 | 0.93 | 0.79 | 87.5 | 0 |
| anxdep_724 | 0.78 | 0.74 | 87.5 | 0 |
| anxdep_511 | 0.91 | 0.62 | 87.5 | 0 |
| anxdep_675 | 0.83 | 0.42 | 87.5 | 0 |
| anxdep_509 | 0.87 | 0.02 | 87.5 | 0 |
| cont_468 | 0.64 | 0.72 | 87.5 | 0 |
| alc_416 | 0.8 | 0.53 | 87.5 | 0 |
| sui_28 | 0.94 | 0.67 | 87.5 | 0 |
| cont_730 | 0.66 | 0.77 | 87.5 | 0 |
| sui_754 | 0.89 | 0.73 | 87.5 | 0 |
| sui_732 | 0.85 | 0.85 | 87.5 | 2 |
| alc_549 | 0.77 | 0.67 | 87.5 | 0 |
| alc_185 | 0.55 | 0.64 | 87.5 | 2 |
| alc_278 | 0.8 | 0.72 | 87.5 | 0 |
| cont_292 | 0.79 | 0.58 | 87.5 | 0 |
| anxdep_7 | 0.91 | 0.7 | 87.5 | 0 |
| anxdep_762 | 0.83 | 0.71 | 87.5 | 0 |
| cont_555 | 0.66 | 0.64 | 87.5 | 0 |
| sui_34 | 0.71 | 0.62 | 87.5 | 0 |
| anxdep_379 | 0.92 | 0.77 | 87.5 | 0 |
| sui_61 | 0.87 | 0.47 | 87.5 | 0 |
| anxdep_82 | 0.85 | 0.87 | 87.5 | 0 |
| sui_149 | 0.89 | 0.79 | 87.5 | 0 |
| anxdep_62 | 0.81 | 0.74 | 87.5 | 0 |
| cont_642 | 0.81 | 0.42 | 87.5 | 0 |
| cont_745 | 0.78 | 0.88 | 87.5 | 20.4 |
| anxdep_65 | 0.84 | 0.71 | 87.5 | 0 |
| anxdep_77 | 0.76 | 0.44 | 87.5 | 0 |
| cont_135 | 0.61 | 0.51 | 87.5 | 0 |
| sui_649 | 0.73 | 0.32 | 87.5 | 0 |
| alc_493 | 0.91 | 0.78 | 85.7 | 2.1 |
| sui_276 | 0.73 | 0.75 | 85.7 | 0 |
| cont_487 | 0.68 | 0.36 | 85.7 | 0 |
| anxdep_148 | 0.91 | 0.72 | 85.7 | 0 |
| sui_319 | 0.86 | 0.7 | 85.7 | 0 |
| sui_637 | 0.81 | 0.79 | 85.7 | 0 |
| alc_402 | 0.93 | 0.85 | 85.7 | 0 |
| sui_294 | 0.93 | 0.81 | 85.7 | 0 |
| cont_499 | 0.78 | 0.36 | 85.7 | 0 |
| anxdep_603 | 0.87 | 0.69 | 85.7 | 0 |
| alc_396 | 0.57 | 0.48 | 85.7 | 0 |
| cont_167 | 0.73 | 0.45 | 85.7 | 0 |
| alc_640 | 0.97 | 0.8 | 85.7 | 27.1 |
| alc_563 | 0.83 | 0.77 | 85.7 | 0 |
| alc_227 | 0.44 | 0.78 | 85.7 | 0 |
| alc_777 | 0.81 | 0.77 | 85.7 | 0 |
| alc_498 | 0.71 | 0.74 | 85.7 | 0 |
| alc_165 | 0.8 | 0.77 | 85.7 | 0 |
| cont_106 | 0.83 | 0.75 | 85.7 | 0 |
| alc_285 | 0.85 | 0.68 | 85.7 | 0 |
| alc_414 | 0.77 | 0.66 | 85.7 | 0 |
| anxdep_542 | 0.55 | 0.66 | 85.7 | 0 |
| alc_417 | 0.88 | 0.64 | 85.7 | 0 |
| sui_550 | 0.71 | 0.2 | 85.7 | 41.7 |
| alc_508 | 0.57 | #N/A | 85.7 | 0 |
| cont_323 | 0.66 | 0.45 | 85.7 | 0 |
| alc_146 | 0.78 | 0.55 | 85.7 | 0 |
| alc_264 | 0.75 | 0.54 | 85.7 | 0 |
| alc_632 | 0.88 | 0.76 | 85.7 | 0 |
| alc_582 | 0.92 | 0.82 | 85.7 | 0 |
| anxdep_385 | 0.76 | 0.74 | 85.7 | 20.8 |
| anxdep_635 | 0.75 | 0.42 | 85.7 | 0 |
| alc_362 | 0.94 | 0.74 | 85.7 | 0 |
| alc_431 | 0.85 | 0.83 | 85.7 | 0 |
| sui_310 | 0.84 | 0.74 | 85.7 | 2.1 |
| cont_648 | 0.68 | 0.17 | 85.7 | 0 |
| anxdep_166 | 0.88 | 0.37 | 85.7 | 0 |
| anxdep_597 | 0.84 | 0.35 | 85.7 | 0 |
| anxdep_33 | 0.78 | 0.67 | 85.7 | 0 |
| alc_434 | 0.79 | 0.73 | 85.7 | 0 |
| alc_688 | 0.57 | 0.38 | 85.7 | 0 |
| sui_758 | 0.83 | 0.86 | 85.7 | 0 |
| cont_32 | 0.71 | 0.47 | 85.7 | 0 |
| cont_720 | 0.91 | 0.87 | 85.7 | 2.1 |
| sui_312 | 0.91 | 0.77 | 85.7 | 0 |
| cont_42 | 0.78 | 0.47 | 85.7 | 0 |
| cont_543 | 0.92 | 0.77 | 83.9 | 0 |
| alc_49 | 0.66 | 0.34 | 83.9 | 0 |
| anxdep_346 | 0.57 | 0.69 | 83.9 | 6.4 |
| alc_697 | 0.77 | 0.74 | 83.9 | 0 |
| cont_448 | 0.68 | 0.6 | 83.9 | 0 |
| alc_513 | 0.7 | 0.66 | 83.9 | 0 |
| cont_418 | 0.28 | 0.61 | 83.9 | 0 |
| alc_764 | 0.87 | 0.41 | 83.9 | 0 |
| alc_178 | 0.66 | 0.43 | 83.9 | 0 |
| alc_109 | 0.87 | 0.78 | 83.9 | 2.1 |
| sui_530 | 0.87 | 0.77 | 83.9 | 2.1 |
| anxdep_328 | 0.61 | 0.56 | 83.9 | 0 |
| sui_170 | 0.85 | 0.66 | 83.9 | 0 |
| alc_115 | 0.68 | 0.5 | 83.9 | 0 |
| sui_665 | 0.73 | 0.43 | 83.9 | 0 |
| anxdep_618 | 0.8 | 0.88 | 83.9 | 0 |
| alc_274 | 0.79 | 0.9 | 83.9 | 0 |
| alc_154 | 0.67 | 0.73 | 83.9 | 0 |
| anxdep_561 | 0.82 | 0.85 | 83.9 | 0 |
| anxdep_650 | 0.78 | 0.78 | 83.9 | 0 |
| cont_289 | 0.82 | 0.55 | 83.9 | 0 |
| anxdep_602 | 0.58 | 0.52 | 83.9 | 0 |
| anxdep_38 | 0.86 | 0.79 | 83.9 | 2.1 |
| cont_155 | 0.78 | 0.25 | 83.9 | 0 |
| alc_192 | 0.77 | 0.54 | 83.9 | 0 |
| sui_756 | 0.93 | 0.77 | 83.9 | 0 |
| alc_126 | 0.79 | 0.86 | 82.1 | 0 |
| cont_354 | 0.76 | 0.45 | 82.1 | 0 |
| sui_609 | 0.89 | 0.78 | 82.1 | 0 |
| alc_601 | 0.75 | 0.27 | 82.1 | 0 |
| cont_685 | 0.62 | 0.32 | 82.1 | 0 |
| alc_341 | 0.79 | 0.64 | 82.1 | 0 |
| anxdep_463 | 0.55 | 0.6 | 82.1 | 0 |
| anxdep_471 | 0.79 | 0.58 | 82.1 | 0 |
| anxdep_249 | 0.81 | 0.67 | 82.1 | 0 |
| sui_445 | 0.9 | 0.81 | 82.1 | 0 |
| cont_432 | 0.89 | 0.89 | 82.1 | 0 |
| sui_98 | 0.88 | 0.72 | 82.1 | 0 |
| sui_569 | 0.86 | 0.76 | 82.1 | 0 |
| cont_410 | 0.76 | 0.58 | 82.1 | 0 |
| sui_600 | 0.86 | 0.89 | 82.1 | 0 |
| alc_17 | 0.91 | 0.91 | 82.1 | 6.5 |
| alc_610 | 0.76 | 0.9 | 82.1 | 6.5 |
| alc_406 | 0.89 | 0.87 | 82.1 | 0 |
| cont_663 | 0.76 | 0.42 | 82.1 | 0 |
| cont_541 | 0.79 | 0.84 | 82.1 | 0 |
| sui_398 | 0.87 | 0.77 | 80.4 | 0 |
| alc_30 | 0.9 | 0.84 | 80.4 | 0 |
| anxdep_709 | 0.67 | 0.59 | 80.4 | 0 |
| anxdep_351 | 0.84 | 0.68 | 80.4 | 0 |
| alc_112 | 0.49 | 0.63 | 80.4 | 0 |
| sui_171 | 0.84 | 0.32 | 80.4 | 0 |
| alc_234 | 0.83 | 0.84 | 80.4 | 0 |
| alc_321 | 0.84 | 0.83 | 80.4 | 0 |
| anxdep_480 | 0.59 | 0.49 | 80.4 | 8.9 |
| alc_244 | -0.03 | 0.24 | 80.4 | 0 |
| alc_231 | 0.67 | 0.36 | 80.4 | 0 |
| alc_133 | 0.87 | 0.86 | 80.4 | 0 |
| sui_641 | 0.94 | 0.88 | 80.4 | 0 |
| cont_297 | 0.63 | 0.35 | 80.4 | 0 |
| anxdep_11 | 0.88 | 0.79 | 80.4 | 0 |
| sui_421 | 0.83 | 0.67 | 80.4 | 0 |
| alc_481 | 0.65 | 0.32 | 80.4 | 0 |
| anxdep_514 | 0.89 | 0.53 | 80.4 | 0 |
| anxdep_200 | 0.78 | 0.79 | 80.4 | 2.2 |
| anxdep_20 | 0.74 | 0.46 | 80.4 | 0 |
| cont_391 | 0.8 | 0.53 | 78.6 | 0 |
| anxdep_467 | 0.44 | 0.66 | 78.6 | 0 |
| anxdep_408 | 0.86 | 0.87 | 78.6 | 0 |
| anxdep_9 | 0.81 | 0.64 | 78.6 | 0 |
| alc_386 | 0.9 | 0.86 | 78.6 | 0 |
| sui_93 | 0.91 | 0.68 | 78.6 | 2.3 |
| alc_510 | 0.72 | 0.5 | 76.8 | 0 |
| anxdep_627 | 0.88 | 0.7 | 76.8 | 0 |
| anxdep_271 | 0.83 | 0.81 | 76.8 | 0 |
| anxdep_226 | 0.68 | 0.7 | 76.8 | 0 |
| cont_125 | 0.77 | 0.63 | 76.8 | 0 |
| sui_141 | 0.87 | 0.88 | 76.8 | 0 |
| sui_384 | 0.79 | 0.67 | 76.8 | 0 |
| sui_607 | 0.65 | 0.76 | 76.8 | 0 |
| cont_4 | 0.87 | 0.72 | 76.8 | 0 |
| cont_287 | 0.85 | 0.8 | 76.8 | 0 |
| alc_331 | 0.62 | 0.49 | 75 | 0 |
| alc_769 | 0.34 | 0.2 | 75 | 0 |
| anxdep_451 | 0.91 | 0.52 | 75 | 0 |
| anxdep_41 | 0.76 | 0.61 | 75 | 0 |
| alc_780 | 0.9 | 0.66 | 75 | 0 |
| cont_196 | 0.5 | 0.46 | 75 | 0 |
| sui_327 | 0.93 | 0.81 | 75 | 2.4 |
| alc_86 | 0.84 | 0.57 | 75 | 0 |
| cont_366 | 0.81 | 0.87 | 75 | 4.8 |
| sui_357 | 0.87 | 0.67 | 75 | 0 |
| sui_545 | 0.85 | 0.87 | 73.2 | 0 |
| sui_643 | 0.8 | 0.44 | 73.2 | 0 |
| sui_534 | 0.9 | 0.89 | 73.2 | 14.6 |
| alc_173 | 0.65 | 0.6 | 73.2 | 0 |
| alc_69 | 0.58 | 0.65 | 73.2 | 2.4 |
| alc_121 | 0.74 | 0.87 | 73.2 | 0 |
| cont_765 | 0.83 | 0.89 | 73.2 | 7.3 |
| cont_584 | 0.7 | 0.68 | 73.2 | 0 |
| sui_693 | 0.73 | 0.44 | 73.2 | 0 |
| sui_401 | 0.67 | 0.5 | 73.2 | 4.9 |
| sui_446 | 0.87 | 0.82 | 73.2 | 0 |
| alc_381 | 0.76 | 0.77 | 71.4 | 0 |
| anxdep_407 | 0.91 | 0.89 | 71.4 | 0 |
| alc_64 | 0.84 | 0.85 | 71.4 | 0 |
| cont_427 | 0.75 | 0.75 | 71.4 | 0 |
| anxdep_352 | 0.59 | 0.82 | 71.4 | 0 |
| anxdep_117 | 0.84 | 0.66 | 71.4 | 0 |
| sui_476 | 0.75 | 0.65 | 71.4 | 0 |
| alc_218 | 0.73 | 0.76 | 69.6 | 10.3 |
| alc_420 | 0.88 | 0.85 | 69.6 | 0 |
| cont_163 | 0.75 | 0.49 | 69.6 | 0 |
| cont_638 | 0.59 | 0.8 | 69.6 | 0 |
| sui_71 | 0.65 | 0.22 | 66.1 | 0 |
| sui_206 | 0.79 | 0.87 | 66.1 | 0 |
| alc_252 | 0.62 | 0.59 | 66.1 | 0 |
| sui_75 | 0.77 | 0.79 | 66.1 | 0 |
| anxdep_522 | 0.85 | 0.8 | 66.1 | 0 |
| cont_240 | 0.81 | 0.66 | 66.1 | 0 |
| alc_644 | 0.68 | 0.62 | 64.3 | 0 |
| cont_747 | 0.39 | 0.15 | 64.3 | 0 |
| cont_778 | 0.74 | 0.56 | 64.3 | 0 |
| anxdep_72 | 0.81 | 0.82 | 64.3 | 0 |
| sui_761 | 0.68 | 0.76 | 62.5 | 0 |
| alc_194 | 0.73 | 0.16 | 62.5 | 0 |
| anxdep_103 | 0.6 | 0.43 | 62.5 | 0 |
| alc_412 | 0.86 | 0.76 | 60.7 | 0 |
| cont_128 | 0.67 | 0.67 | 60.7 | 0 |
| alc_224 | 0.83 | 0.78 | 60.7 | 0 |
| anxdep_137 | 0.82 | 0.52 | 60.7 | 0 |
| sui_604 | 0.9 | 0.88 | 60.7 | 0 |
| alc_531 | 0.83 | 0.64 | 58.9 | 0 |
| alc_466 | 0.86 | 0.35 | 58.9 | 3 |
| cont_382 | 0.93 | 0.88 | 58.9 | 0 |
| anxdep_590 | 0.89 | 0.8 | 58.9 | 0 |
| alc_538 | 0.85 | 0.49 | 57.1 | 0 |
| anxdep_291 | 0.78 | 0.86 | 57.1 | 0 |
| anxdep_186 | 0.81 | 0.48 | 57.1 | 0 |
| alc_87 | 0.82 | 0.77 | 55.4 | 0 |
| sui_419 | 0.94 | 0.94 | 55.4 | 3.2 |
| alc_279 | 0.89 | 0.79 | 55.4 | 0 |
| sui_393 | 0.58 | 0.56 | 53.6 | 0 |
| cont_110 | 0.66 | 0.24 | 53.6 | 0 |
| sui_262 | 0.84 | 0.84 | 51.8 | 0 |
| anxdep_315 | 0.81 | 0.73 | 51.8 | 3.4 |
| alc_565 | 0.77 | 0.81 | 50 | 0 |
| alc_502 | 0.71 | 0.72 | 50 | 0 |
| anxdep_211 | 0.91 | 0.8 | 50 | 0 |
| anxdep_465 | 0.77 | 0.41 | 50 | 0 |
| alc_330 | 0.9 | 0.48 | 48.2 | 0 |
| sui_12 | 0.89 | 0.75 | 48.2 | 0 |
| alc_722 | 0.87 | 0.75 | 46.4 | 0 |
| anxdep_507 | 0.88 | 0.84 | 46.4 | 0 |
| cont_107 | 0.75 | 0.51 | 46.4 | 0 |
| alc_435 | 0.92 | 0.73 | 44.6 | 0 |
| cont_247 | 0.64 | 0.58 | 44.6 | 0 |
| sui_363 | 0.86 | 0.81 | 44.6 | 0 |
| sui_562 | 0.86 | 0.88 | 44.6 | 0 |
| cont_548 | 0.81 | 0.88 | 44.6 | 0 |
| sui_581 | 0.66 | 0.76 | 44.6 | 0 |
| alc_768 | 0.83 | 0.41 | 44.6 | 0 |
| cont_403 | 0.91 | 0.89 | 44.6 | 0 |
| sui_580 | 0.5 | 0.62 | 42.9 | 0 |
| alc_661 | 0.94 | 0.82 | 42.9 | 0 |
| alc_595 | 0.77 | 0.7 | 42.9 | 0 |
| anxdep_647 | 0.86 | 0.67 | 42.9 | 0 |
| alc_444 | 0.79 | 0.44 | 42.9 | 0 |
| alc_239 | 0.68 | 0 | 41.1 | 0 |
| cont_723 | 0.45 | 0.05 | 41.1 | 0 |
| anxdep_442 | 0.68 | 0.58 | 41.1 | 39.1 |
| anxdep_716 | 0.7 | 0.52 | 39.3 | 0 |
| cont_544 | 0.89 | 0.67 | 39.3 | 0 |
| sui_474 | 0.76 | 0.83 | 39.3 | 0 |
| sui_528 | 0.87 | 0.75 | 39.3 | 0 |
| anxdep_204 | 0.75 | 0.72 | 35.7 | 0 |
| alc_673 | 0.7 | 0.59 | 35.7 | 5 |
| cont_153 | 0.88 | 0.8 | 35.7 | 0 |
| alc_425 | 0.9 | 0.65 | 35.7 | 0 |
| sui_338 | 0.79 | 0.68 | 35.7 | 0 |
| sui_288 | 0.84 | 0.35 | 33.9 | 0 |
| sui_23 | 0.7 | 0.24 | 33.9 | 0 |
| sui_773 | 0.91 | 0.92 | 33.9 | 0 |
| sui_537 | 0.79 | 0.77 | 33.9 | 0 |
| sui_439 | 0.83 | 0.72 | 32.1 | 0 |
| anxdep_399 | 0.79 | 0.53 | 32.1 | 0 |
| alc_710 | 0.63 | 0.8 | 30.4 | 0 |
| anxdep_560 | 0.48 | 0.51 | 30.4 | 0 |
| sui_356 | 0.84 | 0.77 | 30.4 | 0 |
| cont_583 | 0.54 | 0.55 | 30.4 | 0 |
| cont_374 | 0 | #N/A | 30.4 | 0 |
| anxdep_53 | 0.9 | 0.86 | 28.6 | 0 |
| sui_97 | 0.88 | 0.88 | 28.6 | 0 |
| alc_475 | 0.91 | 0.79 | 28.6 | 0 |
| cont_613 | 0.63 | 0.54 | 28.6 | 0 |
| sui_623 | 0.87 | 0.68 | 26.8 | 0 |
| anxdep_438 | 0.91 | 0.87 | 26.8 | 0 |
| anxdep_771 | 0.27 | 0.84 | 25 | 0 |
| alc_368 | 0.47 | 0.2 | 25 | 0 |
| sui_215 | 0.8 | -0.07 | 25 | 0 |
| alc_233 | 0.7 | 0.8 | 23.2 | 0 |
| alc_273 | 0.91 | 0.75 | 21.4 | 0 |
| alc_376 | 0.76 | 0.68 | 21.4 | 16.7 |
| alc_656 | 0.85 | 0 | 21.4 | 16.7 |
| alc_25 | 0.76 | 0.9 | 21.4 | 0 |
| alc_188 | 0.92 | 0.67 | 19.6 | 0 |
| cont_15 | 0.42 | -1.64 | 19.6 | 0 |
| cont_512 | 0.92 | 0.96 | 19.6 | 27.3 |
| cont_314 | 0.71 | 0 | 19.6 | 0 |
| sui_248 | 0.82 | 0.75 | 19.6 | 0 |
| sui_518 | 0.92 | 0.8 | 19.6 | 0 |
| alc_311 | 0.19 | 0.11 | 17.9 | 0 |
| anxdep_536 | 0.59 | 0.11 | 17.9 | 0 |
| cont_350 | 0.79 | -0.03 | 17.9 | 0 |
| sui_701 | 0.72 | 0.64 | 17.9 | 0 |
| anxdep_515 | 0.89 | 0.21 | 16.1 | 11.1 |
| alc_243 | 0.79 | -0.11 | 16.1 | 0 |
| sui_645 | 0.61 | 0.93 | 16.1 | 0 |
| alc_689 | 0.7 | -0.53 | 16.1 | 0 |
| alc_101 | 0.65 | 0.12 | 16.1 | 0 |
| sui_318 | 0.8 | 0.32 | 16.1 | 0 |
| anxdep_461 | 0.53 | 0.61 | 14.3 | 0 |
| anxdep_286 | 0.37 | 0.06 | 14.3 | 0 |
| cont_387 | 0.82 | 0.79 | 14.3 | 0 |
| anxdep_546 | 0.94 | 0.9 | 14.3 | 0 |
| alc_46 | 0.9 | 0.81 | 14.3 | 0 |
| anxdep_766 | 0.86 | 0.93 | 12.5 | 0 |
| anxdep_486 | 0.26 | 0.9 | 12.5 | 0 |
| alc_743 | 0.7 | 0.48 | 12.5 | 0 |
| sui_469 | 0.72 | 0.79 | 12.5 | 0 |
| alc_625 | 0.93 | 0.53 | 10.7 | 0 |
| alc_140 | -0.24 | 0.77 | 10.7 | 0 |
| cont_325 | 0.25 | 0.96 | 10.7 | 0 |
| alc_371 | -4.77 | -0.57 | 10.7 | 0 |
| alc_168 | 0.92 | -1.11 | 10.7 | 0 |
| anxdep_296 | 0.87 | 0.69 | 10.7 | 0 |
| anxdep_268 | -1.03 | 0.76 | 10.7 | 0 |
| cont_198 | 0.83 | #N/A | 10.7 | 0 |
| cont_525 | 0.77 | 0.91 | 10.7 | 0 |
| cont_436 | 0.83 | 0.93 | 8.9 | 0 |
| sui_156 | 0.95 | 0.74 | 8.9 | 0 |
| sui_74 | 0.11 | -0.06 | 8.9 | 0 |
| alc_225 | 0.36 | 0.84 | 8.9 | 0 |
| cont_3 | 0.75 | 0.55 | 8.9 | 0 |
| anxdep_719 | 0.44 | 0.67 | 7.1 | 0 |
| alc_564 | 0.8 | 0 | 7.1 | 0 |
| alc_726 | 0.81 | 0.07 | 7.1 | 0 |
| alc_100 | 0.7 | 0.64 | 7.1 | 0 |
| cont_551 | 0.97 | 0.92 | 7.1 | 0 |
| alc_175 | 0.87 | 0.5 | 7.1 | 0 |
| sui_763 | 0.71 | -9.67 | 7.1 | 0 |
| anxdep_365 | 0.9 | 0.87 | 5.4 | 0 |
| sui_760 | 0.78 | -2.67 | 5.4 | 0 |
| cont_779 | 0.29 | -8 | 5.4 | 0 |
| sui_504 | 0.88 | 0.61 | 5.4 | 0 |
| alc_566 | 0.77 | 0 | 5.4 | 0 |
| alc_718 | 0 | 0.56 | 5.4 | 0 |
| anxdep_309 | 0.78 | -0.57 | 5.4 | 0 |
| anxdep_591 | 0.78 | 0.59 | 5.4 | 0 |
| alc_639 | 0.18 | 0.44 | 5.4 | 0 |
| sui_378 | -2.5 | 0.91 | 5.4 | 0 |
| sui_369 | 0 | 0.67 | 5.4 | 0 |
| cont_78 | 0.78 | -2.67 | 3.6 | 0 |
| alc_775 | -5 | #N/A | 3.6 | 0 |
| cont_704 | #DIV/0! | 0 | 3.6 | 0 |
| alc_696 | -0.62 | -2.67 | 3.6 | 0 |
| anxdep_237 | 0.6 | 0.96 | 3.6 | 0 |
| alc_347 | -1.25 | #DIV/0! | 3.6 | 0 |
| anxdep_676 | 0.83 | 0.83 | 3.6 | 0 |
| anxdep_304 | 0.7 | 0.99 | 3.6 | 50 |
| anxdep_658 | #DIV/0! | -5.33 | 3.6 | 0 |
| cont_322 | #DIV/0! | 0 | 3.6 | 0 |
| cont_389 | 0.5 | #N/A | 3.6 | 0 |
| sui_477 | 0.62 | #N/A | 3.6 | 0 |
| alc_19 | #N/A | #N/A | 1.8 | 0 |
| anxdep_752 | #N/A | #N/A | 1.8 | 0 |
| anxdep_353 | #N/A | #N/A | 1.8 | 0 |
| cont_36 | #N/A | #N/A | 1.8 | 0 |
| anxdep_29 | #N/A | #N/A | 1.8 | 0 |
| alc_440 | #N/A | #N/A | 1.8 | 0 |
| sui_557 | #N/A | #N/A | 1.8 | 0 |
| anxdep_751 | #N/A | #N/A | 1.8 | 0 |

| *Acronyms: PA (Positive Affect), NA (Negative Affect).* |
| --- |
| *[1] Raw alphas: reliability estimate based upon the covariance of items.* |
| *[2] Compliance is defined as the proportion of momentary assessments completed divided by the maximum number of momentary assessments allowed by the design, i.e., 56.* |
| *[3] Percentage of assessments identified as careless (i.e., with a time per item response equal or below 1 second and no variation in responses) among the total of assessments completed by the individual.* |
